# Phylogenomics of Prokaryotic Ribosomal Proteins

**DOI:** 10.1371/journal.pone.0036972

**Published:** 2012-05-16

**Authors:** Natalya Yutin, Pere Puigbò, Eugene V. Koonin, Yuri I. Wolf

**Affiliations:** National Center for Biotechnology Information, National Library of Medicine, National Institutes of Health, Bethesda, Maryland, United States of America; Université Paris-Sud, France

## Abstract

Archaeal and bacterial ribosomes contain more than 50 proteins, including 34 that are universally conserved in the three domains of cellular life (bacteria, archaea, and eukaryotes). Despite the high sequence conservation, annotation of ribosomal (r-) protein genes is often difficult because of their short lengths and biased sequence composition. We developed an automated computational pipeline for identification of r-protein genes and applied it to 995 completely sequenced bacterial and 87 archaeal genomes available in the RefSeq database. The pipeline employs curated seed alignments of r-proteins to run position-specific scoring matrix (PSSM)-based BLAST searches against six-frame genome translations, mitigating possible gene annotation errors. As a result of this analysis, we performed a census of prokaryotic r-protein complements, enumerated missing and paralogous r-proteins, and analyzed the distributions of ribosomal protein genes among chromosomal partitions. Phyletic patterns of bacterial and archaeal r-protein genes were mapped to phylogenetic trees reconstructed from concatenated alignments of r-proteins to reveal the history of likely multiple independent gains and losses. These alignments, available for download, can be used as search profiles to improve genome annotation of r-proteins and for further comparative genomics studies.

## Introduction

The ribosome, the molecular machine for protein biosynthesis, is the hallmark of cellular life forms [1]. The high resolution atomic structure of the ribosome [2]–[4] is considered among the pinnacles of the achievements of the structural biology [5]. In addition to three or four essential, highly conserved rRNA molecules, the large (50S) and small (30S) ribosomal subunits contain over 50 distinct ribosomal (r) proteins that interact with the rRNAs and with one another. Among these, 34 r-proteins are universally conserved in the three domains of cellular life (bacteria, archaea and eukaryotes); 33 r-proteins are shared between archaea and eukaryotes to the exclusion of bacteria; 23 r-proteins are bacteria-specific, 1 r-protein is archaea-specific and 11 r-proteins are eukaryotes-specific [6]. In addition, we included in our analysis three recently discovered ribosomal proteins that appear to be specific for the Sulfolobales/Desulfurococcales branch of archaea [7]. In bacteria and archaea, genes encoding r-proteins are organized in genomic clusters that include several partially conserved operons and are often called ribosomal superoperons [8], [9]. Systematic analysis of gene neighborhoods shows that ribosomal superoperons are the largest partially conserved gene arrays in bacterial and archaeal genomes [10], [11].

The r-proteins are nearly universal, typically highly conserved and highly expressed which makes them particularly relevant for deep phylogenetic analysis and related evolutionary studies [8], [12]–[18].

However, some of the r-protein genes are difficult targets for automatic annotation in sequenced genomes because they are short and compositionally biased. Problems in r-proteins annotation inspired the RibAlign project [19] that, however, has been *de facto* abandoned by the end of 2011. Here we report a comprehensive reannotation of r-proteins in genomes of 995 bacteria and 87 archaea and discuss trends of their distribution across the different branches of life and patterns in their evolution.

## Results

### Data collection

**Figure 1:**
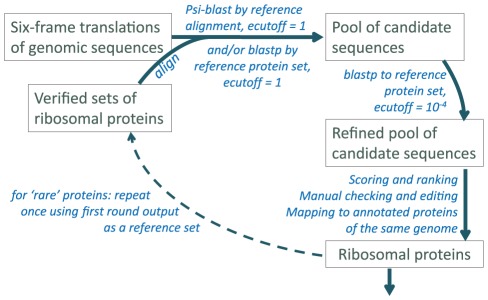
Overall scheme of the procedure.

In order to derive comprehensive sets of bacterial and archaeal r-proteins, we developed a two-step procedure that is schematically shown in Figure 1 (see Methods for details). Briefly, position-specific scoring matrices (PSSMs) for 56 bacterial and 71 archaeal r-proteins [6], [7] (Table S1) were used to screen completely sequenced prokaryotic genomes (Table S2) translated in six frames. Lists of candidate r-proteins were further refined by manually checking for false positives and false negatives and fixing likely frameshifts. In bacteria, 52,692 primary r-proteins and 1,274 additional paralogs were identified; for archaea, the numbers were 5,412 and 26, respectively (Table S3). Among these proteins 796 (1.5%) bacterial and 45 (0.74%) archaeal open reading frames (ORFs) were not annotated as proteins in the Refseq database; 447 (0.85%) bacterial and 67 (1.2%) archaeal ORFs were misannotated (Table S4).

The dataset is available at <ftp://ftp.ncbi.nih.gov/pub/wolf/_suppl/ribo/>.

### Phyletic distribution and evolution of r-proteins in bacteria

Of the 56 bacterial r-proteins, 44 were found to be strictly ubiquitous in 995 bacterial genomes. Six proteins, L19p, L31p, L34p, L36p, L9p and S16p, were missing in only one to three genomes. Another six proteins, L7ae, L25p, L30p, S21p, S22p and S31e (also known as Thx or plastid-specific ribosomal protein 4), were identified in a much smaller fraction of bacteria (the same 6 proteins were marked as non-ubiquitous in bacteria by Lecompte et al. [6]).

**Figure 2:**
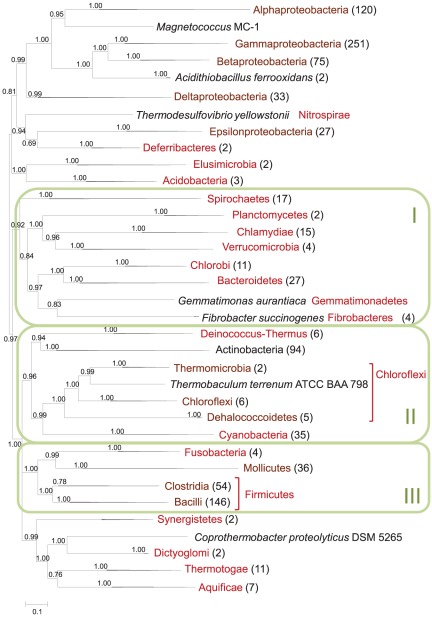
Bacterial phylogenetic tree reconstructed from a concatenated alignment of 50 nearly ubiquitous r-proteins. Green boxes denoted as I, II, and III mark three putative “megaphyla” discussed in the text. Branches having bootstrap support values less than 0.5 were collapsed.

To map the phyletic patterns of r-proteins onto the consensus phylogeny of the bacterial ribosome, we reconstructed a phylogenetic tree from a concatenated alignment of 50 nearly ubiquitous r-proteins from 995 bacteria (Figure 2 and File S1). The tree was rooted using the Modified Mid-Point Rooting (MMPR) procedure [20]. This topology of the r-protein tree is generally compatible with the commonly accepted bacterial taxonomy (<http://www.ncbi.nlm.nih.gov/Taxonomy>) but several notable deviations exist:

- The proteobacterial branch includes phyla *Deferribacteres* and *Nitrospirae*. This topology is in an agreement with the recent phylogenetic study based on gene order comparison which suggests that the *Deferribacteres* is a group phylogenetically proximal to the *Proteobacteria* and *Nitrospirae* [21]. In the r-protein tree, these two phyla are grouped with *Epsilonproteobacteria*;
- *Magnetococcus* MC-1 currently assigned to unclassified *Proteobacteria* appears as the deepest branch of *Alphaproteobacteria* (cf. [22], [23]);
- *Acidithiobacillus ferrooxidans* currently assigned to *Gammaproteobacteria*, is placed in the root of *Gamma*- and *Betaproteobacteria* group [24];
- *Elusimicrobia* and *Acidobacteria* form a sister group to *Proteobacteria*;
- *Fibrobacteres*/*Acidobacteria* group is not supported (cf. [15]);
- an unclassified bacterium *Thermobaculum terrenum* ATCC BAA 798 is placed in the *Chloroflexi* phylum in a basal position of *Thermomicrobia* class;
- *Coprothermobacter proteolyticus* DSM 5265 assigned to *Firmicutes* is placed in the *Dictyoglomia*-*Thermotogae*-*Aquificae* group, sister to *Dictyoglomia* [25].

In addition, the r-protein tree includes, with strong bootstrap support, three deep unifications of bacterial (super)phyla that are not part of the current taxonomy. These major branches of the r-protein tree consist of:

- *Spirochaetes*, the PVC (*Planctomycetes*, *Verrucomicrobia*, *Chlamydia*) superphylum, the *Chlorobi-Bacteroidetes* group, *Gemmatimonadetes* and *Fibrobacteres* (denoted I in Figure 2) [15], [26];
- *Deinococcus-Thermus* group, *Actinobacteria*, *Chloroflexi* and *Cyanobacteria* (II) [27];
- *Firmicutes* (including *Mollicutes* a.k.a. *Tenericutes*) and *Fusobacteria* (III)

Some of these deep relationships, in particular the unification of *Spirochaetes* and *Chlamydia*, and of the *Deinococcus-Thermus* group with *Actinobacteria* and *Cyanobacteria*, have been suggested by various phylogenomic approaches in previous studies performed with limited sets of available genomes [12], [28], [29]. There are many biases that can affect the topology of phylogenetic trees, especially when deep branches are concerned, and detailed statistical analysis of the global tree is beyond the scope of the present work. Nevertheless, the recurrent appearance of the “megaphyla” in trees constructed with different approaches [12], [15], [28], [29] and on expanding sets of genomes suggests that further, in-depth analysis of the relationships between the respective bacterial phyla is warranted.

**Figure 3:**
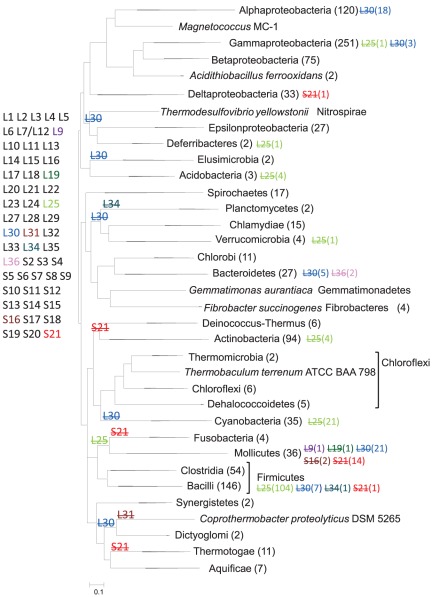
Phyletic patterns of r-proteins that are placed in the last common ancestor of Bacteria by Dollo parsimony. Proteins having full phyletic pattern are listed in black font. Losses are marked by a strikethrough font. Numbers in parentheses following taxonomic group names represent number of species in that group. Numbers in parentheses following r-protein names represent number of species on that branch that have lost this r-protein.

**Figure 4:**
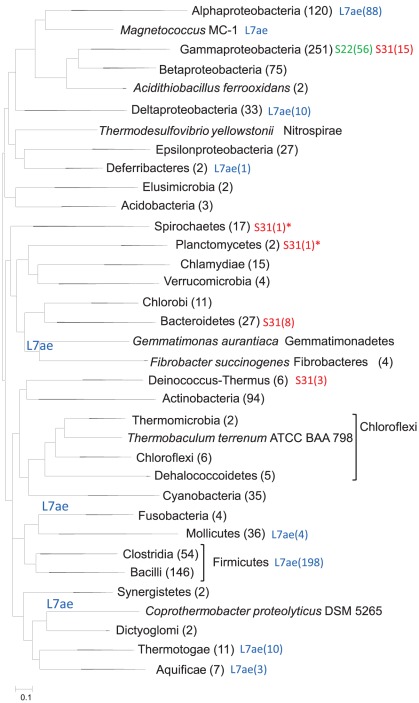
Phyletic distribution of non-ubiquitous bacterial r-proteins that according to the parsimony reconstruction do not appear to be ancestral. Numbers in parentheses following taxonomic group names represent number of species in that group. Numbers in parentheses following r-protein names represent number of species on that branch that have this r-protein. L7ae on branch means all genomes of this branch included in the dataset have this r-protein. Asterisks point on two phyla where S31 protein has been found in genomes that were not included in the dataset.

The phylogenetic tree of concatenated r-proteins was used to map the phyletic patterns of non-ubiquitous bacterial r-proteins (Figures 3 and 4). The S21, L25, and L30 proteins are missing in 131, 162, and 145 genomes, respectively. Dollo parsimony analysis of these patterns suggests several independent losses of each of these proteins during bacterial evolution; it should be noted that due to the relatively shallow location of the majority of the inferred losses, these results are largely robust to the reconstruction method and the exact position of the root.

Figure 4 represents the phyletic distribution of non-ubiquitous bacterial r-proteins that according to the parsimony reconstruction do not appear to be ancestral. The S22 protein, also known as SRA protein (stationary-phase-induced ribosome-associated protein [30]), was identified only in some enterobacteria including all species of *Citrobacter*, *Enterobacter*, *Escherichia*, *Klebsiella*, *Salmonella*, and three of the 7 *Shigella* species.

The S31e protein, also known as Thx peptide in *Thermus*, is a small protein with a central alpha-helix deeply embedded into the 16S RNA core of the small subunit [31], This protein is present in all *Thermales*, some *Gammaproteobacteria* and all *Bacteroidetes* except *Capnocytophaga ochracea* DSM 7271. Surprisingly, although this peptide is present in chloroplast ribosomes [32], it was not found in any *Cyanobacteria*. Analysis of partially sequenced and draft genomes (running tblastn and blastp searches against the nr database) demonstrated the presence of this peptide in two additional phyla, namely *Spirochaetes* (*Spirochaeta thermophila* DSM 6192) and *Planctomycetes* (*Isosphaera pallida* ATCC 43644) (File S2). The other 17 *Spirochaetes* present in the data set used in this study (3 *Treponema*, 6 *Leptospira*, and 8 *Borrelia* species) as well as *Planctomycetes* (2 *Pirellula* species) lack the Thx peptide. The Thx peptide gene is located downstream of the 5S ribosomal RNA gene in *Spirochaeta thermophila* DSM 6192 and downstream of the r-protein S20 in *Thermus thermophilus*, but in other species the location of this gene is distinct from the position of ribosomal genes.

The L30 protein is missing in both cyanobacteria and chloroplasts; L25 that is missing in chloroplasts is missing in 21 of the 35 cyanobacteria.

The L7ae protein was identified in *Aquificae*, *Deferribacteres*, *Firmicutes*, *Fusobacteria*, *Gemmatimonadetes*, *Alpha-* and *Deltaproteobacteria*, *Synergistetes*, and *Thermotogae*. These bacterial proteins often contain a YlxR domain (cd00279) [33] that is located upstream of their L7ae domain. The L7ae gene is duplicated in many *Bacilli* (98 species) and in both *Synergistetes*. Although this protein is relatively rare, its scattered phyletic pattern suggests that it might have been present in the last common ancestor of bacteria.

### Paralogous r-proteins in Bacteria

We identified a total of 1,274 r-protein paralogs in 536 of the 995 analyzed bacterial genomes. Some phyla encode no paralogous r-proteins: *Acidobacteria*, *Elusimicrobia*, *Deferribacteres*, *Epsilonproteobacteria*, *Fibrobacteres*, *Fusobacteria*, *Planctomycetes*, *Chlamydiae/Verrucomicrobia*, *Thermotogae*, and *Coprothermobacter*. Conversely, genes for some r-proteins seem to never duplicate: L9, L20, L27, L35, S6, S20, S22, S31. *Actinobacteria* and *Firmicutes* encode the largest numbers of r-protein paralogs (∼2.5 per genome on average; File S3). Strikingly, *Leptospira borgpetersenii* serovar *Hardjo-bovis* L550 has 26 r-proteins duplicated; *Bartonella bacilliformis* KC583 has 16. Other six *Leptospira* species and seven *Bartonella* species present in the analyzed data set lack these massive duplications. All r-protein paralogs in these two species are identical at the nucleotide level which might indicate a very recent duplication or a genome assembly artifact. Overall, 74 paralogous r-proteins are identical or nearly identical (>97% identical amino acids) copies of the top-ranked paralog (the one most similar to the query profile) from the same genome, whereas 571 of them differ significantly (<50% identical amino acids) (File S3) Notably, all highly diverged paralogs are r-proteins containing various forms of the Zn-binding motif [34]–[36]. The presence of divergent homologs of r-proteins in a genome might imply presence of paralogs resulting from ancient duplications, xenologs acquired via HGT and/or accelerated evolution of more recent paralogous copies. It has been shown previously that paralogs of several r-proteins, in particular those that differ by the presence or absence of Zn-coordinating cysteines, are functionally different, i.e., different paralogs are incorporated into the ribosome depending on the physiological conditions such as zinc ion concentration [34], [36], [37]. Possibly, functional differentiation might be involved in retention of other duplicated r-proteins as well.

### Distribution of ribosomal protein genes across bacterial genome partitions

In 68 of the 995 analyzed bacterial genomes, r-protein genes are distributed across two or more genome partitions. In some cases, paralogous proteins are encoded in different chromosomes or plasmids. For example, *Rhizobium leguminosarum* bv. *trifolii* WSM1325 possesses four paralogous copies of S21 protein gene. Two of these are located on the major chromosome (NC_012850, 4.8 Mbp) whereas two others are on different plasmids (NC_012853, 516 Kp and NC_012854, 295 Kbp). In other cases, r-protein genes are present in a single copy but are spread across genome partitions. In *Paracoccus denitrificans* PD1222, 42 ribosomal protein genes are located on Chromosome 1 (NC_008686, 2.9 Mbp), whereas the remaining 12 genes are on Chromosome 2 (NC_008687, 1.7 Mbp). In *Shewanella baltica* OS155, 6 of its single-copy r-protein genes are located on a 17 Kbp plasmid NC_009037 (Table S5).

### Phyletic distribution and evolution of r-proteins in Archaea

**Figure 5:**
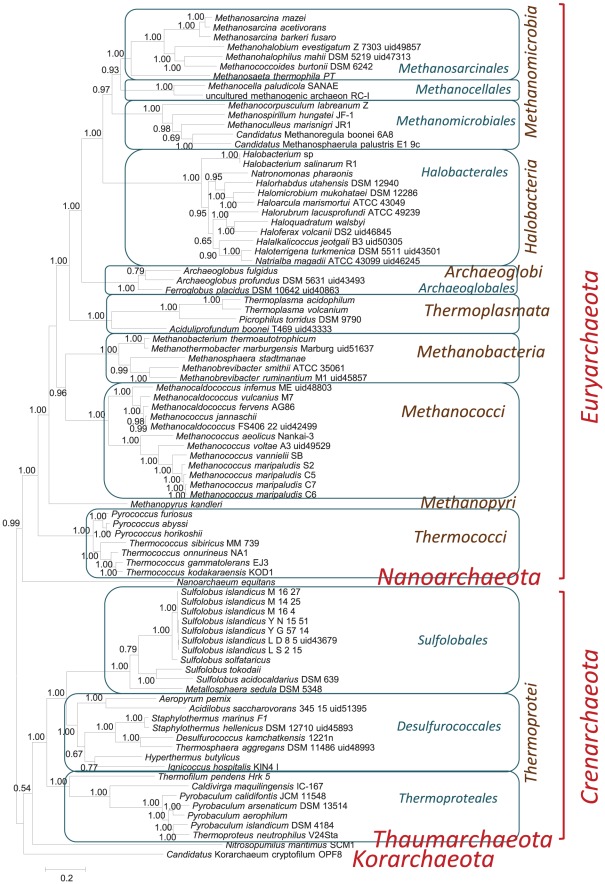
Archaeal phylogenetic tree reconstructed from a concatenated alignment of 56 ubiquitous r-proteins.

Among the 71 archaeal r-proteins (68 from [6] and 3 from [7]), 56 are ubiquitous in the analyzed set of 87 genomes. Proteins S27ae, L18ae, and L30e are nearly ubiquitous (found in 84, 78 and 72 genomes, respectively); and 9 r-proteins (L14e, L34e, S26e, S30e, S25e, L41e, L13e, L35ae, and L38e) are present in 53 or fewer archaeal genomes. We used the 56 ubiquitous archaeal r-proteins to reconstruct a phylogenetic tree from a concatenated alignment (Figure 5, File S4). The tree was rooted using the MMPR procedure [20]). The tree topology is compatible with the current archaeal taxonomy (<http://www.ncbi.nlm.nih.gov/Taxonomy/>), with the following exceptions:

- unclassified uncultured methanogenic archaeon RC-1 confidently groups with *Methanocella* (*Methanomicrobia, Euryarchaeota*);
- unclassified *Aciduliprofundum boonei* T469 groups with *Thermoplasmatales* (*Euryarchaeota*);
- *Acidilobus saccharovorans* 345-15, classified as *Acidilobales* [38], groups with *Desulfurococcales* (*Thermoprotei*, *Crenarchaeota*);

These three unconvential clades have been reported in a recent phylogenetic study with which our present results closely agree [39].

As is the case with bacteria, the results of phylogenetic analysis of r-proteins are compatible with some “superphyla”, in particular, the “TACK” superphylum that encompasses *Thaumarchaeota*, *Crenarchaeota* and *Korarchaeota* as well as the recently proposed phylum *Aigarchaeota* [40]. Well resolved internal structure appears also within *Euryarchaeota* and includes in particular a strongly supported clade (putative superphylum) that encompasses the majority of known mesophilic euryarchaeota (*Methanomicrobia* and *Halobacteria*) [41]–[43].

**Figure 6:**
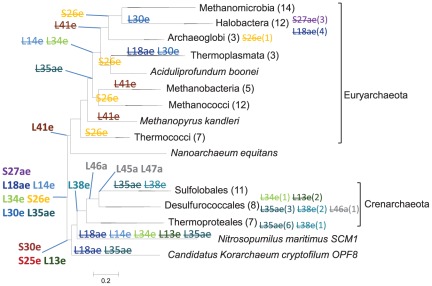
Phyletic distribution of twelve non-ubiquitous archaeal r-proteins. Numbers in parentheses and strikethrough font mean the same as on Figure 3.

The tree was used to map phyletic patterns of non-ubiquitous archaeal r-proteins (Figure 6). As with bacteria, several probable independent losses of proteins L41e, S30e, L18ae, L13e, L35ae, L38e were detected; in particular, evolution of L41e has not been reconstructed previously because this small protein was missed in many genomes. Five r-proteins, L41e, L38e, S30e, S25e, and L13e were inferred to be missing in the last common ancestor of the extant archaea by Dollo parsimony. The origin of L41e was mapped to the last common ancestor of *Eury*- and *Nanoarchaeota*, origin of L38e to the last common ancestor of *Crenarchaeota*, and origin of L30e, S25e, and L13e to the last common ancestor of *Cren*-, *Thaum*-, and *Korarchaeota*. However, taking into account the presence of these proteins in eukaryotes might suggest other evolutionary scenarios (see below). Three recently identified r-poteins [7] show a narrow phyletic range: L45a and L 47a have been detected only in *Sulfolobales*, whereas L46a has been found only in *Sulfolobales* and *Desulfurococcales* (all but *Igniococcus hospitalis* KIN4 I).

A recent study of mitochondrial r-proteins [18] also has briefly addressed the gain/loss pattern of archaeal r-proteins and concluded that archaeal ribosomes have probably undergone multiple independent losses and that the last common ancestor of archaea possessed a more complex ribosome than any of the extant archaea species. Furthermore, in agreement with the results reported here, this study has concluded that the ribosomes of different archaea have lost several r-proteins that are shared between archaea and eukaryotes but not those shared between archaea and bacteria (see File S5 for a detailed comparison).

### Paralogy and distribution of archaeal r-protein genes across genome partitions

Generally r-proteins in archaeal genomes are much less prone to form paralogous families. In archaea there are 26 paralogs, altogether; 17 of them in various *Halobacteria*; 12 out of these are the second paralogs of S10p (Table S3). An overwhelming majority of archaeal ribosomal protein genes in this study are located on the major chromosomes. Four paralogs in *Halobacteria* are located on minor partitions (plasmids) and include two S17e paralogs in *Haloterrigena turkmenica* DSM_5511, an S17e paralog in *Haloarcula marismortui* ATCC 43049 and an S14 paralog in *Natrialba magadii* ATCC 43099.

### Universally conserved r-proteins and the origin of eukaryotes

**Figure 7:**
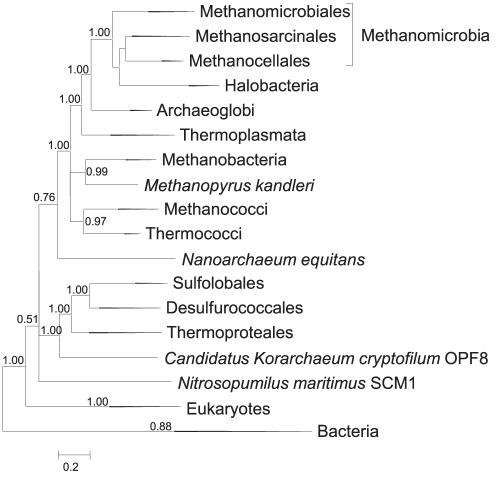
Three-domain phylogenetic tree reconstructed from a concatenated alignment of 32 universal r-proteins. Branches having bootstrap support values less than 0.5 were collapsed.

We reconstructed a ML tree using a concatenated alignment of 32 r-proteins that are conserved in bacteria, archaea and eukaryotes (Figure 7, File S6). The tree that included all 87 archaeal species, 10 representative eukaryotic species and all 995 bacterial species (the latter were used as an outgroup to root the tree) places eukaryotes as the sister group to archaea. This “classical” [44], [45] topology has been obtained previously with a concatenated set of 29 r-proteins (4,571 positions, 121 genomes) [46]. In the trees involving subsampling of species eukaryotes often formed a clade with *Cren*-, *Thaum*- and *Korarchaeota* to the exclusion of *Eury*- and *Nanoarchaeota*. Such position was obtained as the consensus in RAxML analysis of systematic subsamples of archaeal, bacterial and eukaryotic sequences (non-parametric bootstrap support value of 85%, File S7); in a FastTree analysis of a sample with 80 bacterial representatives (FastTree branch support value of 0.85, Table S6 and File S7) and in an analysis of a 100-species subsample (70 bacteria, 20 archaea and 10 eukaryotes) using FastTree (non-parametric bootstrap support value of 54%, File S7) and RAxML (non-parametric bootstrap support value of 82%, File S7). The latter results generally agree with the recently proposed origin of eukaryotes from the TACK superphylum of archaea [40] but analysis of the phyletic patterns of archaeal r-proteins suggests a more complex evolutionary scenario. Remarkably, all five archaeal r-proteins that are not reconstructed as ancestral in archaea are present in eukaryotes. The distribution of one of these lineage-specific r-proteins in archaea (L41e) does not fit the TACK scenario, whereas that of L38e is compatible only with *Crenarchaeota* being the sister group to eukaryotes (Figure 6). These anomalies might suggest either the ancestral provenance of these proteins, with subsequent loss in several archaeal phyla, or a history of gene exchange between ancient archaeal lineages including the putative archaeal ancestor of eukaryotes. This observation agrees with the proposed origin of eukaryotes from a complex, possibly transient archaeal form [47], [48].

## Discussion

In the present work, we compiled a comprehensive collection of bacterial and archaeal r-protein sequences, multiple alignments and PSSMs that can be used both for genome annotation and for a variety of phylogenomic analyses. Numerous r-proteins were identified that remained unannotated, primarily because of their small size, or misannotated in sequenced genomes. Some preliminary phylogenomic results are presented. Despite the overall high evolutionary conservation, for several bacterial and archaeal r-proteins, multiple lineage-specific losses as well as gains were identified. The r-protein genes show a low level of paralogy (geometric mean of 1.02 paralogs per r-protein in bacteria and 1.01 in archaea compared to 1.63 in all nearly universalgenes, see Methods), conceivably due to selective pressure on maintenance of the unitary stoichiometry of r-proteins in the ribosome, the effect known as gene dosage balance [49]–[52]. However, multiple duplications of several r-protein genes were detected, particularly in bacteria. A substantial fraction of these duplications are highly diverged and are likely to possess distinct functions as demonstrated for r-proteins that differ by the presence of absence of a cluster of Zn-coordinating amino acids. Although no special effort was made to eliminate cases of HGT, concatenated r-protein sequences yield robust phylogenetic tree topologies that are compatible with the monophyly of the established phyla. Furthermore, the phylogenetic trees of r-proteins contain several major branches that might correspond to bacterial and archaeal super(mega)phyla. Although any proposals on bacterial and archaeal taxonomy are beyond the scope of this work, further assessment of the validity of these large, deeply branching groups by detailed phylogenomic analysis will be of substantial interest. The results of phylogenetic analysis of r-proteins is generally compatible with the origin of the eukaryotic ribosome from the TACK superphylum of archaea but additionally suggests a complex ancestral archaeal form which encoded all r-proteins that are non-ubiquitous in extant archaea.

## Methods

### Bacterial and archaeal genomes

Genomic data for 995 bacterial and 87 archaeal completely sequenced genomes were retrieved from NCBI Genomes database <ftp://ftp.ncbi.nlm.nih.gov/genomes/Bacteria/> in October 2010 (Table S2). Each genome was conceptually translated in six frames using the corresponding genetic code table. A set of individual ORFs with minimum length of 16 amino acids spanning the range from the first start codon to the first in-frame stop codon was generated from each frame.

### The r-protein set

The set of r-proteins used in this study was essentially the same as in [6]. Ribosomal protein S1p was excluded from the list because of its varied domain architecture and the ubiquity of S1-like domains in a wide variety of RNA-associated proteins unrelated to the ribosome [53]. NCBI COG [54] and arCOG [55] databases were used as sources of initial sets of 56 bacterial and 68 archaeal r-proteins respectively (Table S1). Initial sets of three novel archaeal r-proteins identified by Marquez et al [7] were retrieved using PSI-BLAST [56] searches against the nr database. NCBI protein cluster PRK10057 was used as the initial set for ribosomal protein S22; the initial set for ribosomal protein S31e (a.k.a. THX peptide [31]) was created by PSI-BLAST starting from S31 protein of *Thermus thermophilus* HB8 (TTHA1396). Initial sets were aligned using MUSCLE [57] and used as position-specific scoring matrices (PSSM) in PSI-BLAST [56] searches.

### Search for r-proteins

The ribosomal protein PSSMs were ran against the translated genome databases using PSI-BLAST [56] with the e-value cutoff of 1, collecting a pool of candidate sequences. This pool was refined by reverse BLASTP against the set of initial sequences with the e-value cutoff of 10^−4^. Sequences that passed this threshold were aligned with the corresponding initial alignment; lowest-scoring matches were manually curated; newly identified r-proteins were added to the initial sets. For r-proteins missing in more than 20% of species the reverse BLASTP run against the updated set of confirmed r-proteins was repeated with the e-value cutoff of 10^−2^ instead of 10^−4^, followed by manual verification (see Figure 1). Final sets of r-proteins were mapped to annotated proteins in the same genomes where possible.

### Phylogenetic analysis

All sets of r-proteins were aligned using MUSCLE program [57]. Alignments for 50 bacterial r-proteins (all but S21, S22, L25, L30, S31, and L7ae), filtered to contain positions with less than 50% of gap characters and concatenated producing a 6,127- position alignment. A ML tree was constructed using FastTree program [58] with WAG evolutionary model and discrete gamma model with 20 rate categories). A 7,843-position concatenated alignment of 56 archaeal r-proteins that were present in all of 87 archaeal genomes was used to reconstruct the trees using the same procedure. A phylogenetic tree for a 4,226-position concatenated alignment of 32 universal r-proteins (Table S1, except L30p and L7ae) from all archaea and selected bacteria and eukaryotic species (Table S6) was constructed in the same manner.

Additionally, the optimal amino acid evolution model (LG+G) was selected for the alignment of 32 universal r-proteins using the ProtTest program [59]. This model was used for phylogenetic reconstructions with taxon-sampled alignments using the RAxML program [60] (see File S7 for details).

### Phylogenomic reconstruction of gene gains and losses

Mapping of gene gains and losses to the phylogenetic trees was produced using the Dollo parsimony analysis implemented in DOLLOP program of the PHYLIP package [61].

### Number of paralogs

To avoid the statistical bias due to uneven sampling in the course of genome sequencing we chose 383 representative genomes [62] with at least 500 protein-coding genes. Normally a single representative of the genus with the largest genome was selected; the genus *Shigella* was merged with *Escherichia* and for *Escherichia* and *Bacillus* the ‘model’ genomes of *E. coli* str. K-12 substr. MG1655 and *B. subtilis* subsp. *subtilis* str. 168 were added. For this set of genomes we computed the geometric mean of the number of ribosomal proteins per genome of each kind, separately for bacteria and archaea. For comparison we selected 158 COGs [54] that were present in >90% of these genomes (i.e. nearly universal) and computed the geometric mean of the number of proteins per genome within the dataset.

## Supporting Information

Table S1 List of r-proteins analyzed in this study.
(XLS)

Table S2 List of bacterial and archaeal genomes analyzed in this study.
(XLS)

Table S3 Phyletic distribution of ribosomal proteins collected in this study.
(XLS)

Table S4 List bacterial and archaeal ribosomal proteins that were missing or misannotated in Refseq database.
(XLS)

Table S5 Distribution of bacterial r-proteins among genome partitions.
(XLS)

Table S6 Representative genomes for archaea, bacteria and eukaryotes.
(XLS)

File S1 Newick-formatted tree shown on Figure 2.
(TXT)

File S2 List of bacterial genomes having Thx peptide and multiple alignment of bacterial Thx peptides.
(PDF)

File S3 Distribution of paralogous r-proteins in bacteria.
(PDF)

File S4 Newick-formatted tree shown on Figure 5.
(TXT)

File S5 Detailed comparison of of the pattern of archaeal r-protein gain and loss with Desmond et al., 2011 [18].
(PDF)

File S6 Newick-formatted tree shown on Figure 7.
(TXT)

File S7 Supporting information on phylogenetic analysis.
(PDF)
