# Supplementary material for: Phylogenomics of Prokaryotic Ribosomal Proteins
Source: PLoS One. 2012 May 16;7(5):e36972. doi: 10.1371/journal.pone.0036972 (PMC3353972; doi:10.1371/journal.pone.0036972)
Supplement: File S2 — List of bacterial genomes having Thx peptide and multiple alignment of bacterial Thx peptides. (PDF) [file pone.0036972.s008.pdf]

Multiple alignment of bacterial THX peptide

```
                      *         20         *        40         *        60         *        80         *
Xylella_fa : MGKGDRKTTKGKRYNASYGNARPHSISKA-----------PVDAAAPLSNRRAPKAQS-PRTVVKKTVAKAI------------------------- :  60
Xanthcmona : MGKGDRKTAKGKRYNSSYGNSRSHAVSKV-----------VVGAAAPVAKKGVVKAPA-KKAVAKKSVAKAG------------------------- :  60
Xanthcmona : MGKGDRKTAKGKRYNASYGNSRSHAASKV-----------VVGAAAPVAKKGVVKAPV-KKAVAKKTVAKAS------------------------- :  60
Xanthcmona : MGKGDRKTAKGKRYNASYGNSRSHAVSKV-----------VVGAAAPVAKKGVVKAPA-KKAVAKKTVAKAS------------------------- :  60
Xanthcmona : MGKGDRKTAKGKRYNASYGNARSHSVSKV-----------AVGAAAPVAKKSVVKAPAVKKAVTKKTVAKAG------------------------- :  61
Stenotroph : MGKGDRKTAKGKRYNASYGNARSHTASKV-----------AVGAAAPVAKKTVAKAPA-KKAVAKKAVAKA-------------------------- :  59
Chitinopha : MGRGDIKTKKGKISNGSFGKARPA-------------------------KPKKAAAAK-AKANA--------------------------------- :  38
Robiginita : MGKGDKKTKRGKIANKSFGARRPRKIRKK----------------TPVEEKINIKGKS--------------------------------------- :  42
Spiroscma_ : MGKGDKKSKRGKISRGSYGKTRPS-------------------------KWNDATYKK-PEADSTNSL----------------------------- :  42
Blattabact : MGKGDKKTRRGKIRNKTYGNLRPN-----------------------------------PKNTKKKKKTN--------------------------- :  35
Flavobacte : MGKGDQKSRRGKVTAGSYGKRRPRKSSSVKNIPVTVMDEDEKKDSKVKVRKEVGYPAGKSENAEPPTDAKPKAAAKPKAEKTEKPAEAKPKAKKTEE :  97
Gramella_f : MGRGDKKTKRGKIAIGTSGKLRPK-------------------------RKKFKIKPT-TLANQDKKELQ--------------------------- :  44
Zunongwang : MGRGDKKTKRGKIAIGTNGKLRPK-------------------------RKKFKVKPT-TLAEQDKKDLQ--------------------------- :  44
Rhodotherm : MGKGDRRTRRGKIWRGTYGKYRPK-------------------------KKKKKQQQE-AAAAAK-------------------------------- :  39
Spirochaet : MGKGDKKSRKGKIWRGTYGNTRPK-------------------------PKNLRRRKR-QQGSSS-------------------------------- :  39
Isosphaera : MGKGDRRTFRGKLSRGSFGKCRPRKPSKK--------------ASKPVETAG-ETAPS-TETASDQVT----------------------------- :  52
Meiothermu : MGKGDRRTRRGKIFRGTYGKYRPR-------------------------KK---------------------------------------------- :  26
Thermus_th : MGKGDRRTRRGKIWRGTYGKYRPR-------------------------KKK--------------------------------------------- :  27
             MG4GD 43 4GK    3 G  R
```

G-proteob.

Bacteroidetes

Isosphaera pallida ATCC 43644 protein:

>gi|320103268|ref|YP_004178859.1| hypothetical protein Isop_1726 [Isosphaera pallida ATCC 43644]

MGKGDRRTFRGKLSRGSFGKCRPRKPSKKASKPVETAGETAPSTETASDQVT

Spirochaeta thermophila DSM 6192 THX Gene (not annotated):

>gb|CP001698.1|:637520-637639 Spirochaeta thermophila DSM 6192, complete genome

ATGGGAAAAGGTGACAAGAAGAGCAGGAAGGGCAAGATCTGGAGAGGCACCTACGGGAATACGAGACCGAAGCCCAAGAAC
CTGCGCAGGCGGAAGCGACAGCAGGGATCCTCCTCGTAG

Spirochaeta thermophila DSM 6192 THX Gene Translation:

MGKGDKKSRKGKIWRGTYGNTRPKPKNLRRRKRQQGSSS

List of genomes (nr database as of Apr 14, 2011) having Thx peptide (S31)				
Marinithermus hydrothermalis DSM 14884	Thermales			
Meiothermus ruber DSM 1279	Thermales			
Meiothermus silvanus DSM 9946	Thermales			
Oceanithermus profundus DSM 14977	Thermales			
Thermus scotoductus SA01	Thermales			
Thermus thermophilus S20-Thx operon (rpsTU genes)	Thermales			
Thermus thermophilus HB27	Thermales			
Thermus thermophilus HB8	Thermales			
Marivirga tractuosa DSM 4126	Bacteroidetes; Cytophagia			
Spirosoma linguale DSM 74	Bacteroidetes; Cytophagia			
Blattabacterium sp. (Blattella germanica) str. Bge	Bacteroidetes; Flavobacteria			
Blattabacterium sp. (Periplaneta americana) str. BPLAN	Bacteroidetes; Flavobacteria			
Cellulophaga algicola DSM 14237	Bacteroidetes; Flavobacteria			
Flavobacteriaceae bacterium 351910	Bacteroidetes; Flavobacteria			
Gramella forsetii KT0803	Bacteroidetes; Flavobacteria			
Maribacter sp. HTCC2170	Bacteroidetes; Flavobacteria			
Robiginitalea biformata HTCC2501	Bacteroidetes; Flavobacteria			
Zunongwangia profunda SM-A87	Bacteroidetes; Flavobacteria			
Chitinophaga pinensis DSM 2588	Bacteroidetes; Sphingobacteria			
Rhodothermus marinus DSM 4252	Bacteroidetes; Sphingobacteria			
Rhodothermus marinus DSM 4252	Bacteroidetes; Sphingobacteria			
Sphingobacterium sp 21	Bacteroidetes; Sphingobacteria			
Pseudoxanthomonas suwonensis 111	[g-proteobacteria]			
Stenotrophomonas maltophilia K279a	[g-proteobacteria]			
Stenotrophomonas maltophilia R5513	[g-proteobacteria]			
uncultured gamma proteobacterium	[g-proteobacteria]			
Xanthomonas albilineans	[g-proteobacteria]			
Xanthomonas axonopodis pv citri str 306	[g-proteobacteria]			
Xanthomonas campestris pv campestris	[g-proteobacteria]			
Xanthomonas campestris pv campestris str 8004	[g-proteobacteria]			
Xanthomonas campestris pv campestris str ATCC 33913	[g-proteobacteria]			
Xanthomonas campestris pv vesicatoria str 8510	[g-proteobacteria]			
Xanthomonas oryzae pv oryzae KACC10331	[g-proteobacteria]			
Xanthomonas oryzae pv oryzae MAFF 311018	[g-proteobacteria]			
Xanthomonas oryzae pv oryzae PXO99A	[g-proteobacteria]			
Xylella fastidiosa 9a5c	[g-proteobacteria]			
Xylella fastidiosa M12	[g-proteobacteria]			
Xylella fastidiosa M23	[g-proteobacteria]			
Xylella fastidiosa subsp fastidiosa GB514	[g-proteobacteria]			
Xylella fastidiosa Temecula1	[g-proteobacteria]			
Isosphaera pallida ATCC 43644	[planctomycetes]			
Spirochaeta thermophila DSM 6192	[spirochetes]			

Multiple alignment of bacterial THX peptide in fasta format

>Xylella_fastidiosa,S31,paralog_number:1,seqscore:1 .1950,prot_gi:15836984,N-longer_than_prot(nt):0,C-longer_than_prot(nt):0.
MGKGDRKTTKGKRYNASYGNARPHSISKA-----------PVDAAAPLSNRRAPKAQS-PRTVVKKTVAKAI-------------------------
>Xylella_fastidiosa_M23,S31,paralog_number:1,seqsco re:1.3245,prot_gi:182682295,N-longer_than_prot(nt):81,C-longer_than_prot(nt):0.
MGKGDRKTAKGKRYNASYGNARPHSISKA-----------SVDAAAPLSNKRAPKAQS-PRTVVKKTVTKAI-------------------------
>Xylella_fastidiosa_Temecula1,S31,paralog_number:1, seqscore:1.3245,prot_gi:28199559,N-longer_than_prot(nt):81,C-longer_than_prot(nt):0.
MGKGDRKTAKGKRYNASYGNARPHSISKA-----------SVDAAAPLSNKRAPKAQS-PRTVVKKTVTKAI-------------------------
>Xylella_fastidiosa_M12,S31,paralog_number:1,seqsco re:1.2129,prot_gi:170730930,N-longer_than_prot(nt):0,C-longer_than_prot(nt):0.
MGKGDRKTAKGKRYNASYGNARPHSISKA-----------SVDAAAPLSNKRAPKAQS-PRTVVKKTVTKAI-------------------------
>Xanthomonas_campestris_B100,S31,paralog_number:1,s eqscore:1.2888,prot_gi:188990901,N-longer_than_prot(nt):27,C-longer_than_prot(nt):0.
MGKGDRKTAKGKRYNSSYGNSRSHAVSKV-----------VVGAAAPVAKKGVVKAPA-KKAVAKKSVAKAG-------------------------
>Xanthomonas_campestris_ATCC_33913,S31,paralog_numb er:1,seqscore:1.2888,prot_gi:21232087,N-longer_than_prot(nt):27,C-longer_than_prot(nt):0.
MGKGDRKTAKGKRYNSSYGNSRSHAVSKV-----------VVGAAAPVAKKGVVKAPA-KKAVAKKSVAKAG-------------------------
>Xanthomonas_campestris_8004,S31,paralog_number:1,s eqscore:1.2886,prot_gi:66767787,N-longer_than_prot(nt):27,C-longer_than_prot(nt):0.
MGKGDRKTAKGKRYNSSYGNSRSHAVSKV-----------VVGAAAPVAKKGVVKAPA-KKAVAKKSVAKAG-------------------------
>Xanthomonas_oryzae_PXO99A,S31,paralog_number:1,seq score:1.3178,prot_gi:188575672,N-longer_than_prot(nt):216,C-longer_than_prot(nt):0.
MGKGDRKTAKGKRYNASYGNSRSHAASKV-----------VVGAAAPVAKKGVVKAPV-KKAVAKKTVAKAS-------------------------
>Xanthomonas_oryzae_KACC10331,S31,paralog_number:1, seqscore:1.3178,prot_gi:58581157,N-longer_than_prot(nt):0,C-longer_than_prot(nt):0.
MGKGDRKTAKGKRYNASYGNSRSHAASKV-----------VVGAAAPVAKKGVVKAPV-KKAVAKKTVAKAS-------------------------
>Xanthomonas_oryzae_MAFF_311018,S31,paralog_number: 1,seqscore:1.3178,prot_gi:84623079,N-longer_than_prot(nt):216,C-longer_than_prot(nt):0.
MGKGDRKTAKGKRYNASYGNSRSHAASKV-----------VVGAAAPVAKKGVVKAPV-KKAVAKKTVAKAS-------------------------
>Xanthomonas_citri,S31,paralog_number:1,seqscore:1. 3146,prot_gi:21243554,N-longer_than_prot(nt):45,C-longer_than_prot(nt):0.
MGKGDRKTAKGKRYNASYGNSRSHAVSKV-----------VVGAAAPVAKKGVVKAPA-KKAVAKKTVAKAS-------------------------
>Xanthomonas_campestris_vesicatoria_85-10,S31,paral og_number:1,seqscore:1.3224,prot_gi:78048543,N-longer_than_prot(nt):45,C-longer_than_prot(nt):0.
MGKGDRKTAKGKRYNASYGNSRSHAVSKV-----------VVGAAAPVAKKGVVKAPA-KKAVAKKTVAKAG-------------------------
>Xanthomonas_albilineans,S31,paralog_number:1,seqsc ore:1.2089,prot_gi:285018824,N-longer_than_prot(nt):78,C-longer_than_prot(nt):0.
MGKGDRKTAKGKRYNASYGNARSHSVSKV-----------AVGAAAPVAKKSVVKAPAVKKAVTKKTVAKAG-------------------------
>Stenotrophomonas_maltophilia_K279a,S31,paralog_num ber:1,seqscore:1.2028,prot_gi:190573450,N-longer_than_prot(nt):0,C-longer_than_prot(nt):0.
MGKGDRKTAKGKRYNASYGNARSHTASKV-----------AVGAAAPVAKKTVAKAPA-KKAVAKKAVAKA--------------------------
>Stenotrophomonas_maltophilia_R551_3,S31,paralog_nu mber:1,seqscore:1.2028,prot_gi:194364994,N-longer_than_prot(nt):0,C-longer_than_prot(nt):0.
MGKGDRKTAKGKRYNASYGNARSHTASKV-----------AVGAAAPVAKKTVAKAPA-KKAVAKKAVAKA--------------------------
>Chitinophaga_pinensis_DSM_2588,S31,paralog_number: 1,seqscore:0.5952,prot_gi:256419933,N-longer_than_prot(nt):21,C-longer_than_prot(nt):0.
MGRGDIKTKKGKISNGSFGKARPA-------------------------KPKKAAAAK-AKANA---------------------------------
>Robiginitalea_biformata_HTCC2501,S31,paralog_numbe r:1,seqscore:0.8062,prot_gi:260063590,N-longer_than_prot(nt):228,C-longer_than_prot(nt):0.
MGKGDKKTKRGKIANKSFGARRPRKIRKK----------------TPVEEKINIKGKS---------------------------------------
>Spirosoma_linguale_DSM_74_uid43413,S31,paralog_num ber:1,seqscore:0.6328,prot_gi:NULL,N-longer_than_prot(nt):NULL,C-longer_than_prot(nt):NULL.
MGKGDKKSKRGKISRGSYGKTRPS-------------------------KWNDATYKK-PEADSTNSL-----------------------------
>Blattabacterium__Blattella_germanica__Bge,S31,para log_number:1,seqscore:0.6309,prot_gi:262340972,N-longer_than_prot(nt):42,C-longer_than_prot(nt):0.
MGKGDKKTRRGKIRNKTYGNLRPN-----------------------------------PKNTKKKKTN----------------------------
>Flavobacteriaceae_bacterium_3519_10,S31,paralog_nu mber:1,seqscore:1.1343,prot_gi:NULL,N-longer_than_prot(nt):NULL,C-longer_than_prot(nt):NULL.
MGKGDQKSRRGKVTAGSYGKRRPRKSSSVKNIPVTVMDEDEKKDSKVKVRKEVGYPAGKSENAEPPTDAKPKAAAKPKAEKTEKPAEAKPKAKKTEE
>Gramella_forsetii_KT0803,S31,paralog_number:1,seqs core:0.6020,prot_gi:120437013,N-longer_than_prot(nt):0,C-longer_than_prot(nt):0.
MGRGDKKTKRGKIAIGTSGKLRPK-------------------------RKKFKIKPT-TLANQDKKELQ---------------------------
>Zunongwangia_profunda_SM_A87_uid48073,S31,paralog_ number:1,seqscore:0.6335,prot_gi:295132697,N-longer_than_prot(nt):9,C-longer_than_prot(nt):0.
MGRGDKKTKRGKIAIGTNGKLRPK-------------------------RKKFKVKPT-TLAEQDKKDLQ---------------------------
>Rhodothermus_marinus_DSM_4252,S31,paralog_number:1 ,seqscore:0.7839,prot_gi:268317217,N-longer_than_prot(nt):57,C-longer_than_prot(nt):0.
MGKGDRRTRRGKIWRGTYGKYRPK-------------------------KKKKKQQQE-AAAAAK--------------------------------
>Spirochaeta_thermophila_DSM_6192 gb|CP001698.1|:637520-637639
MGKGDKKSRKGKIWRGTYGNTRPK-------------------------PKNLRRRKR-QQGSSS--------------------------------
>Isosphaera_pallida_ATCC_43644 gi|320103268|ref|YP_004178859.1| hypothetical protein Isop_1726 [Isosphaera pallida ATCC 43644].
MGKGDRRTFRGKLSRGSFGKCRPRKPSKK--------------ASKPVETAG-ETAPS-TETASDQVT-----------------------------
>Meiothermus_ruber_DSM_1279_uid46661,S31,paralog_nu mber:1,seqscore:0.0000,prot_gi:NULL,N-longer_than_prot(nt):NULL,C-longer_than_prot(nt):NULL.
MGKGDRRTRRGKIFRGTYGKYRPR-------------------------KK--
>Thermus_thermophilus_HB27,S31,paralog_number:1,seq score:0.0000,prot_gi:46199332,N-longer_than_prot(nt):0,C-longer_than_prot(nt):0.
MGKGDRRTRRGKIWRGTYGKYRPR-------------------------KKK---
>Thermus_thermophilus_HB8,S31,paralog_number:1,seqs core:0.0000,prot_gi:55981365,N-longer_than_prot(nt):0,C-longer_than_prot(nt):0.
MGKGDRRTRRGKIWRGTYGKYRPR-------------------------KKK---
