# Supplementary material for: Phylogenomics of Prokaryotic Ribosomal Proteins
Source: PLoS One. 2012 May 16;7(5):e36972. doi: 10.1371/journal.pone.0036972 (PMC3353972; doi:10.1371/journal.pone.0036972)

Number of paralogous ribosomal proteins per genome

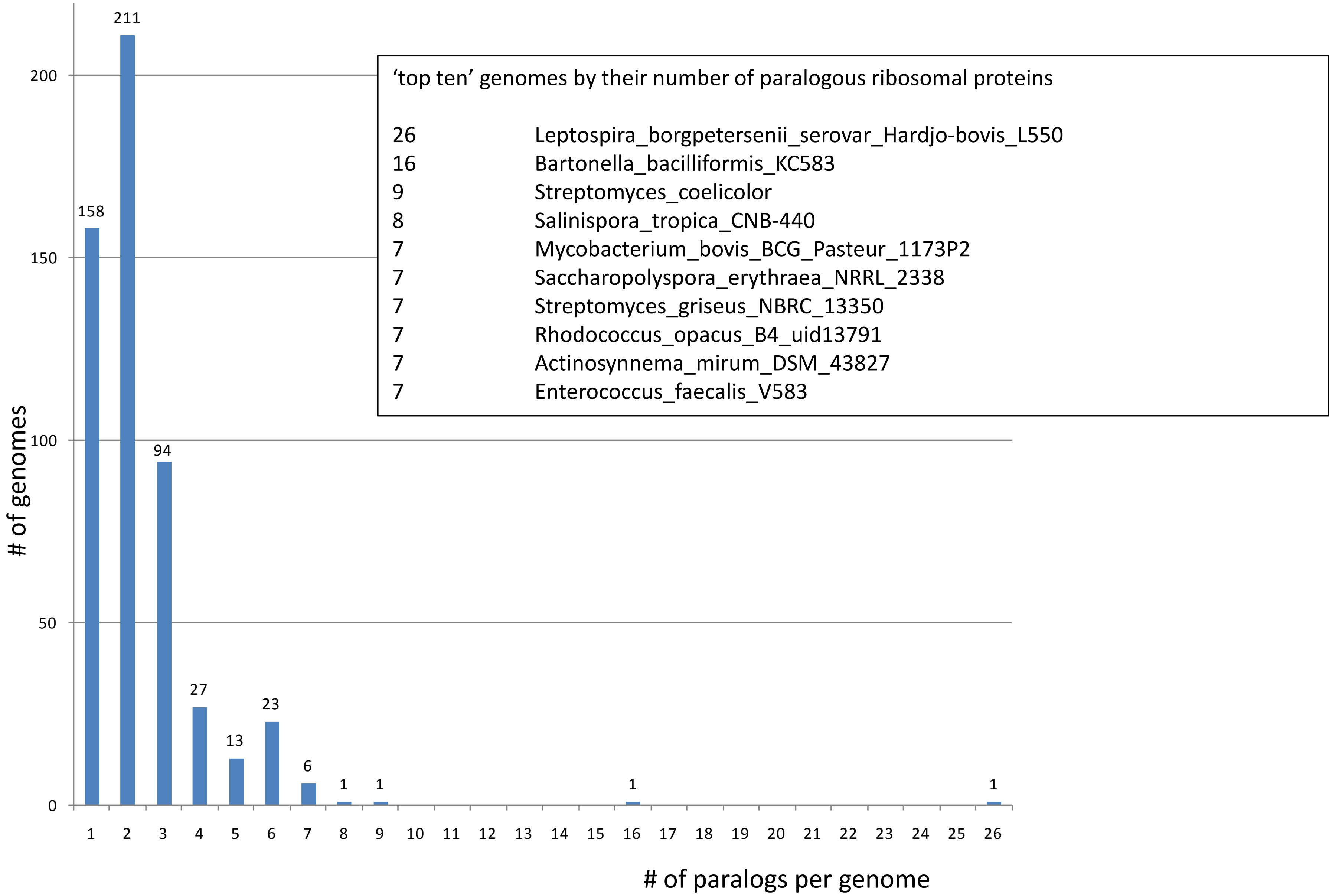

Average number of paralogous ribosomal proteins in different phylogenetic groups

numbers in parentheses represent number of genomes in a group

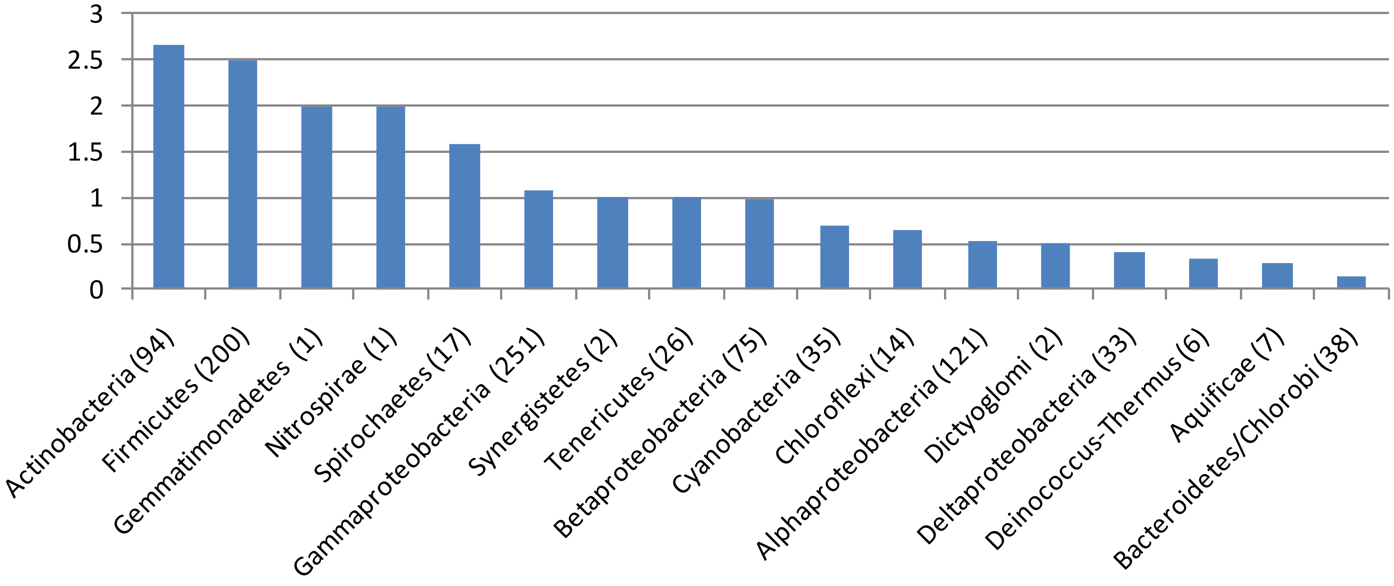

How 2nd paralogs are similar to the 1st ones from the same genome

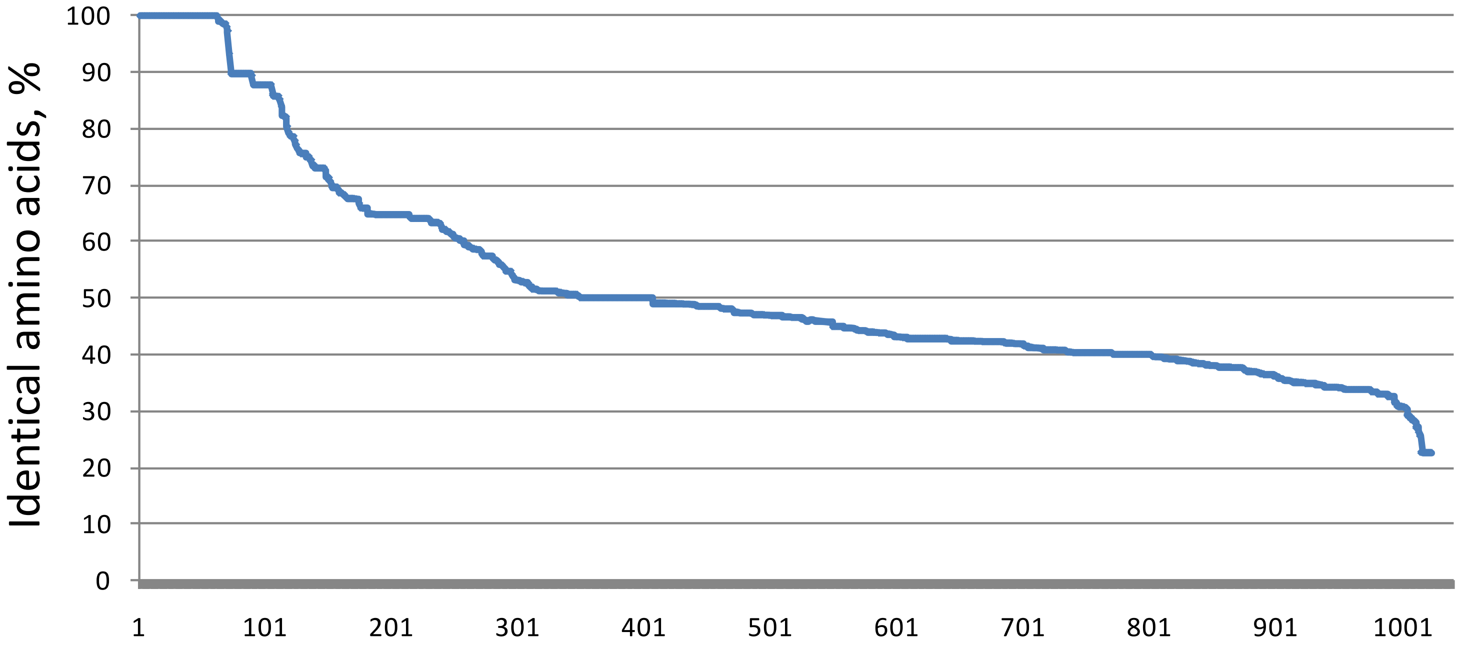

Paralogs of each riboprotein colored by their similarity to the "main" protein of the same genome

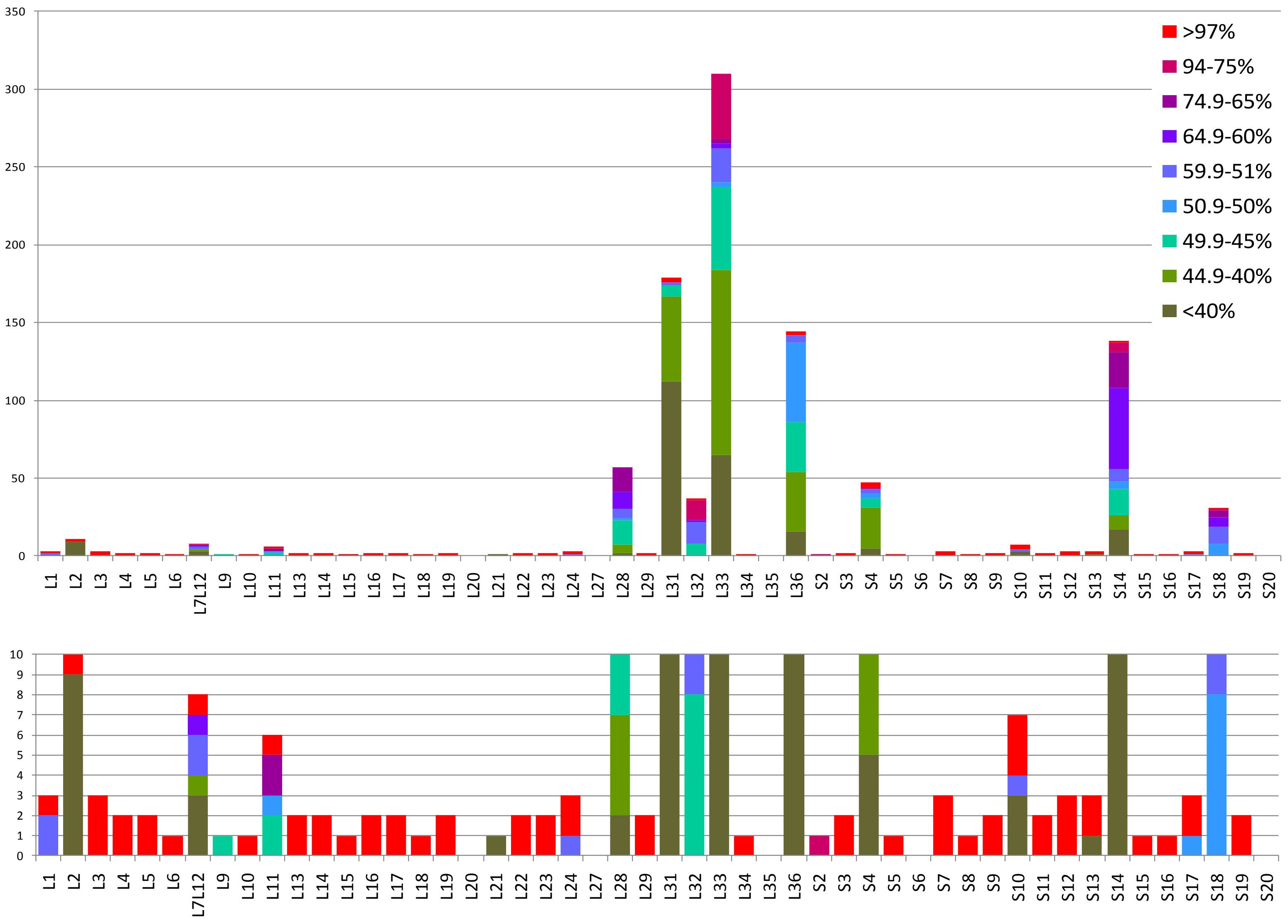

Supplement: File S3 — Distribution of paralogous r-proteins in bacteria. (PDF) [file pone.0036972.s009.pdf]
