# Supplementary material for: Phylogenomics of Prokaryotic Ribosomal Proteins
Source: PLoS One. 2012 May 16;7(5):e36972. doi: 10.1371/journal.pone.0036972 (PMC3353972; doi:10.1371/journal.pone.0036972)
Supplement: File S5 — Detailed comparison of of the pattern of archaeal r-protein gain and loss with Desmond et al., 2011 [18]. (PDF) [file pone.0036972.s011.pdf]

Detailed comparison of the pattern of archaeal r-protein gain and loss reported in Desmond et al., 2011 [1] with our results:

- we used a tree rooted by Euryarchaeota plus Nanoarchaeota; Desmond et al. rooted their tree by Thaumarchaeota that leads to some disparity in root/non-root position of non-universal archaeal r-proteins.
- Based solely on archaeal tree and archaeal pattern (disregarding eukaryotic homologs), we inferred five proteins, L41e, S30e, S25e, L13e, L38e, to several deep branches, but not to the last common ancestor;
- The loss of L18ae, L30e, S27ae by *Halobacteriales* – agreement;
- Losses in Thaumarchaeota – we agree with all but one (Nitrosopumilus does have S24e protein);
- We find all four Nanoarchaeal r-proteins classified as lost by Desmond et al.
- We don't find L14e and L34e in all *Thermoplasmata* so these losses were placed deeper on our tree – not after but before branching *Thermoplasmata* and *A. boonei*;
- Desmond et al. map the loss of S26e to the common ancestor of Eury/Nanoarchaeotes; our study do not confirm this because we did find this protein in two out of three *Archaeoglobi*, *Methanopirus*, and *Nanoarchaeum*.
- We detected r-proteins L29 and L39e in *Korarchaeon*;
- L34e is present in *Caldivirga maquilingensis* (marked as absent in Desmond et al.).

1. Desmond E, Brochier-Armanet C, Forterre P, Gribaldo S (2011) On the last common ancestor and early evolution of eukaryotes: reconstructing the history of mitochondrial ribosomes. Res Microbiol 162: 53-70.
