# Supplementary material for: Phylogenomics of Prokaryotic Ribosomal Proteins
Source: PLoS One. 2012 May 16;7(5):e36972. doi: 10.1371/journal.pone.0036972 (PMC3353972; doi:10.1371/journal.pone.0036972)
Supplement: File S7 — Supporting information on phylogenetic analysis. (PDF) [file pone.0036972.s013.pdf]

Phylogenetic analysis of taxon-sampled alignments.

We used the alignment of 32 ubiquitous r-proteins found in all species in our dataset (995 bacteria, 87 archaea and 10 eukaryotes) and the corresponding tree reconstructed by FastTree program [1] (sites with over 50% of gaps filtered out, WAG evolutionary model, discrete gamma model with 20 rate categories) to find the best-fitting amino acid substitution model. ProtTest program [2] was used to compare different evolutionary models; virus- and mitochondria-specific models were excluded. Following published recommendations[3], we also excluded models involving special treatment for invariant sites. LG substitution model [4] with gamma-distributed site rates (LG+G) outperformed other models and was selected for further analysis. Taking into account alignment-derived amino acid frequencies does not increase the model goodness of fit, partially allaying concerns that the biased amino acid composition of r-proteins distorts the reconstructed phylogeny (see e.g. [5]).

To obtain a dataset of a size amenable to in-depth phylogenetic analysis we classified all organisms in the original dataset into taxonomic lineages: 28 bacterial (*Acidobacteria, Actinobacteria, Alphaproteobacteria, Aquificae*, Bacteroidetes/Chlorobi group, *Betaproteobacteria, Chlamydiae, Chloroflexi, Coprothermobacter, Cyanobacteria, Deferribacteres, Deinococcus-Thermus, Deltaproteobacteria, Dictyoglomi, Elusimicrobia, Epsilonproteobacteria, Fibrobacteres, Firmicutes, Fusobacteria, Gammaproteobacteria, Gemmatimonadetes, Nitrospirae, Planctomycetes, Spirochaetes, Synergistetes, Tenericutes, Thermotogae, Verrucomicrobia*); 14 archaeal (*Archaeoglobi, Desulfurococcales, Halobacteria, Korarchaeota, Methanobacteria, Methanococci, Methanomicrobia, Methanopyri, Nanoarchaeota, Sulfolobales, Thaumarchaeota, Thermococci, Thermoplasmata, Thermoproteales*); 5 eukaryotic (*Alveolata, Euglenozoa, Heterolobosea, Opisthokonta, Viridiplantae*). For the dataset including all three superkingdoms, bacterial classification was further reduced to 3 lineages (*Proteobacteria, Firmicutes*, Other). We randomly sampled one genome from each lineage, extracted the corresponding sequence from the full alignment, removed sites with over 50% of gaps and reconstructed the phylogenetic tree using the RAxML program [3] (rapid hill-climbing mode, LG evolutionary model with gamma-distributed site rates). This procedure was repeated 100 times for each dataset and an extended majority rule consensus tree was reconstructed using the RAxML program.

Bacterial consensus tree topology (Fig. S1, A) differs significantly from the topology obtained with full set of bacterial species using Fasttree. The most notable differences listed in Table S1, A. Archaeal consensus tree topology (Fig. S1, B) differs from the Fasttree topology as listed in Table S1, B. Analysis of the dataset including bacteria, archaea and eukaryotes (Fig. S1, C) places Eukaryotes inside the TACK superphylum, sister to Thaumarchaeota. On this consensus topology (Fig. S1, C), nanoarchaeal branch is placed on the root of archaeal tree, by contrast to its sister-to-Euryarcaeota position on the FastTree tree.

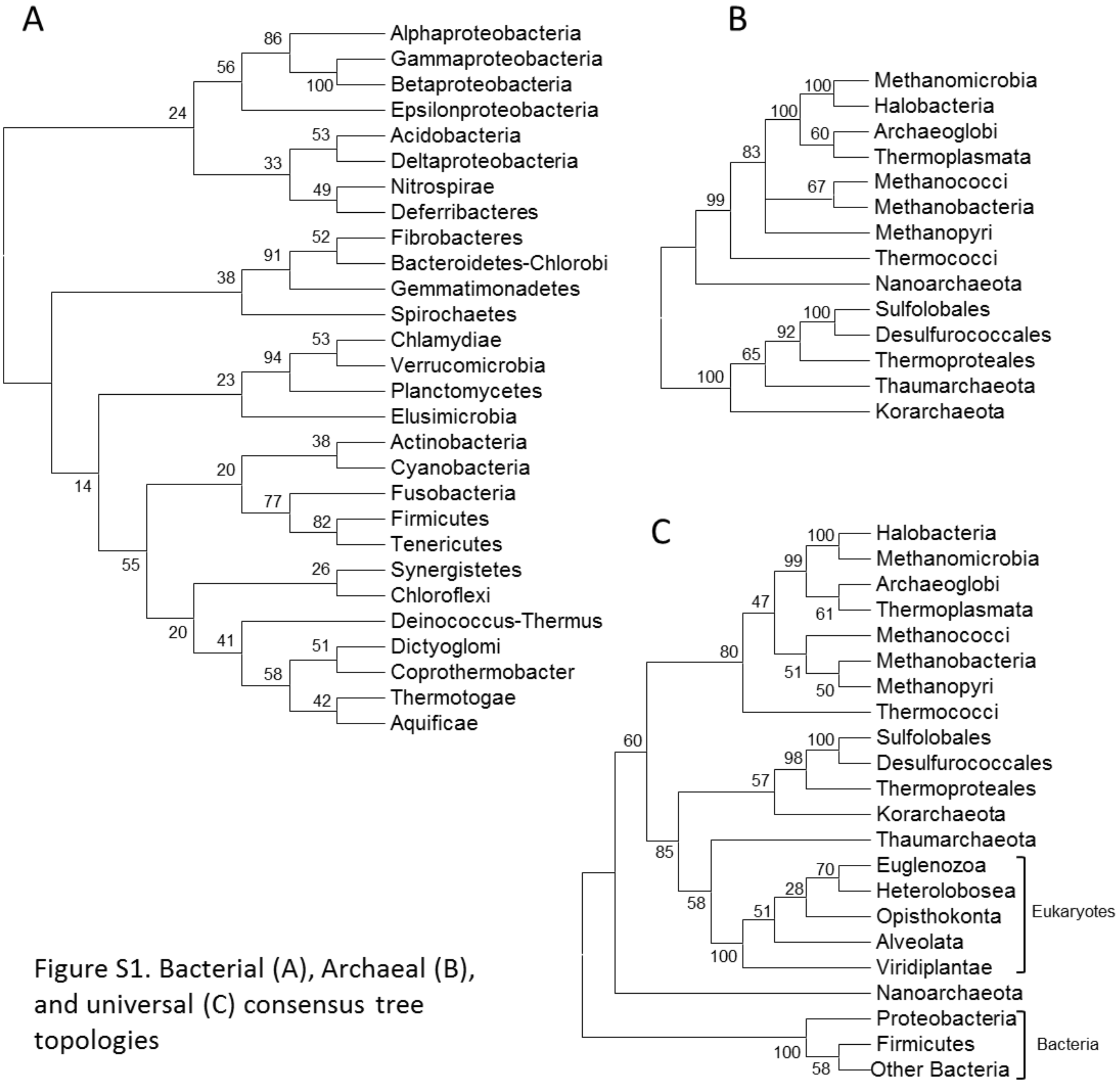

Figure S1. Bacterial (A), Archaeal (B), and universal (C) consensus tree topologies

Table S1. Comparison of Bacterial (A) and Archaeal (B) consensus tree topologies.

A.

Bacterial group	Consensus tree	Fasttree tree
Epsilonproteobacteria	At the root of Alpha/Beta/Gammaproteobacteria	At the root of Alpha/Beta/Gamma/Deltaproteobacteria
Acidobacteria	Sister to Deltaproteobacteria	Sister to Elusimicrobia
Deltaproteobacteria	Sister to Acidobacteria; deeper topology is unresolved	At the root of Alpha/Beta/Gammaproteobacteria
Elusimicrobia	unresolved	Sister to Acidobacteria; Proteobacteria
Deferribacteres, Nitrospirae	Grouping with Proteobacteria is not supported	Group with Proteobacteria
Deinococcus/Thermus and Actinobacteria	Grouping is not supported	Grouped together
Chloroflexi/Cyanobacteria	Grouping is not supported	Grouped together
Spirochaetes	unresolved	Outgroup of PVC (Planctomycetes, Verrucomicrobia, Chlamydia) superphylum, Bacteroidetes/Chlorobi, Gemmatimonadetes and Fibrobacteres
Synergistetes	unresolved	Grouped with Dictyoglomi/Thermotogae/Aquificae

B.

Archaeal group	Consensus tree	Fasttree tree
Thermoplasmata	Sister to Archaeoglobi	Outgroup to Archaeoglobi/Halobacteria/Methanomicrobia
Methanopyri	Unresolved trifurcation of Methanopyri, Methanobacteria/Methanococci, Thermoplasmata/Archaeoglobi/ Halobacteria/Methanomicrobia	Outgroup to Methanobacteria/Methanococci, Thermoplasmata, Archaeoglobi/Halobacteria/Methanomicrobia

Effect of alignment filtering.

To assess the effect of alignment filtering parameters on the phylogeny reconstruction we reconstructed the phylogenetic tree for alignment of 32 r-proteins with removal of sites with 30% and 50% of gap characters with and without additional filtering for homogeneity [6] (homogeneity thresholds of 0.0 and 0.2). Trees were compared using the TOPD software [7]. The split distance in all comparisons didn't exceed 0.1 (i.e. all pairs of trees shared at least 90% of their bipartitions; see TOPD results below). Thus we concluded that the phylogenetic reconstruction of r-proteins is robust to minor variations in alignment filtering.

############### topd 30_0.tre - 30_2.tre #################
* Percentage of taxa in common: 100.0%
* Split Distance [differents/possibles]: 0.0734618916437099 [160 / 2178]
* Split Distance random [differents/possibles]: 0.999853076216712 +/- 0.000 [2177.68 +/- 0.786 / 2178 +/- 0.000]

############### topd 30_0.tre - 50_0.tre #################
* Percentage of taxa in common: 100.0%
* Split Distance [differents/possibles]: 0.0927456382001837 [202 / 2178]
* Split Distance random [differents/possibles]: 0.999788797061524 +/- 0.000 [2177.54 +/- 0.974 / 2178 +/- 0.000]

############### topd 30_0.tre - 50_2.tre #################
* Percentage of taxa in common: 100.0%
* Split Distance [differents/possibles]: 0.0817263544536272 [178 / 2178]
* Split Distance random [differents/possibles]: 0.999733700642791 +/- 0.001 [2177.42 +/- 1.106 / 2178 +/- 0.000]

############### topd 30_2.tre - 50_0.tre #################
* Percentage of taxa in common: 100.0%
* Split Distance [differents/possibles]: 0.108356290174472 [236 / 2178]
* Split Distance random [differents/possibles]: 0.999797979797979 +/- 0.000 [2177.56 +/- 0.962 / 2178 +/- 0.000]

############### topd 30_2.tre - 50_2.tre #################
* Percentage of taxa in common: 100.0%
* Split Distance [differents/possibles]: 0.0560146923783288 [122 / 2178]
* Split Distance random [differents/possibles]: 0.999761248852157 +/- 0.000 [2177.48 +/- 0.964 / 2178 +/- 0.000]

############### topd 50_0.tre - 50_2.tre #################
* Percentage of taxa in common: 100.0%
* Split Distance [differents/possibles]: 0.100091827364555 [218 / 2178]
* Split Distance random [differents/possibles]: 0.999807162534435 +/- 0.000 [2177.58 +/- 0.992 / 2178 +/- 0.000]

Archaeal, Bacterial, and Universal consensus trees in Newick format:

(Methanopyri,((Halobacteria,Methanomicrobia)[100],(Thermoplasmata,Archaeoglobi)[60])[100],(((((((Desulfurococcales,Sulfolobales)[100],Thermoproteales)[92],Thaumarchaeota)[65],Korarchaeota)[100],Nanoarchaeota)[99],Thermococci)[83],(Methanobacteria,Methanococci)[67])[41]);

(Aquificae,((((Synergistetes,Chloroflexi)[26],(((((Chlamydiae,Verrucomicrobia)[53],Planctomycetes)[94],Elusimicrobia)[23],((((Fibrobacteres,Bacteroidetes-Chlorobi)[52],Gemmatimonadetes)[91],Spirochaetes)[38],((((Betaproteobacteria,Gammaproteobacteria)[100],Alphaproteobacteria)[86],Epsilonproteobacteria)[56],((Acidobacteria,Deltaproteobacteria)[53],(Nitrospirae,Deferribacteres)[49])[33])[24])[14])[55],(((Tenericutes,Firmicutes)[82],Fusobacteria)[77],(Cyanobacteria,Actinobacteria)[38])[20])[20])[41],Deinococcus-Thermus)[58],(Coprothermobacter,Dictyoglomi)[51])[42],Thermotogae);

(Halobacteria,((((Methanopyri,Methanobacteria)[50],Methanococci)[51],((((((((Euglenozoa,Heterolobosea)[70],Opisthokonta)[28],Alveolata)[51],Viridiplantae)[100],Thaumarchaeota)[58],(((Desulfurococcales,Sulfolobales)[100],Thermoproteales)[98],Korarchaeota)[57])[85],(((B-other,B-firm)[58],B-prot)[100],Nanoarchaeota)[60])[80],Thermococci)[47])[99],(Archaeoglobi,Thermoplasmata)[61])[100],Methanomicrobia);

FastTree phylogeny reconstruction of a sample of Bacteria, Archaea and Eukaryotes:

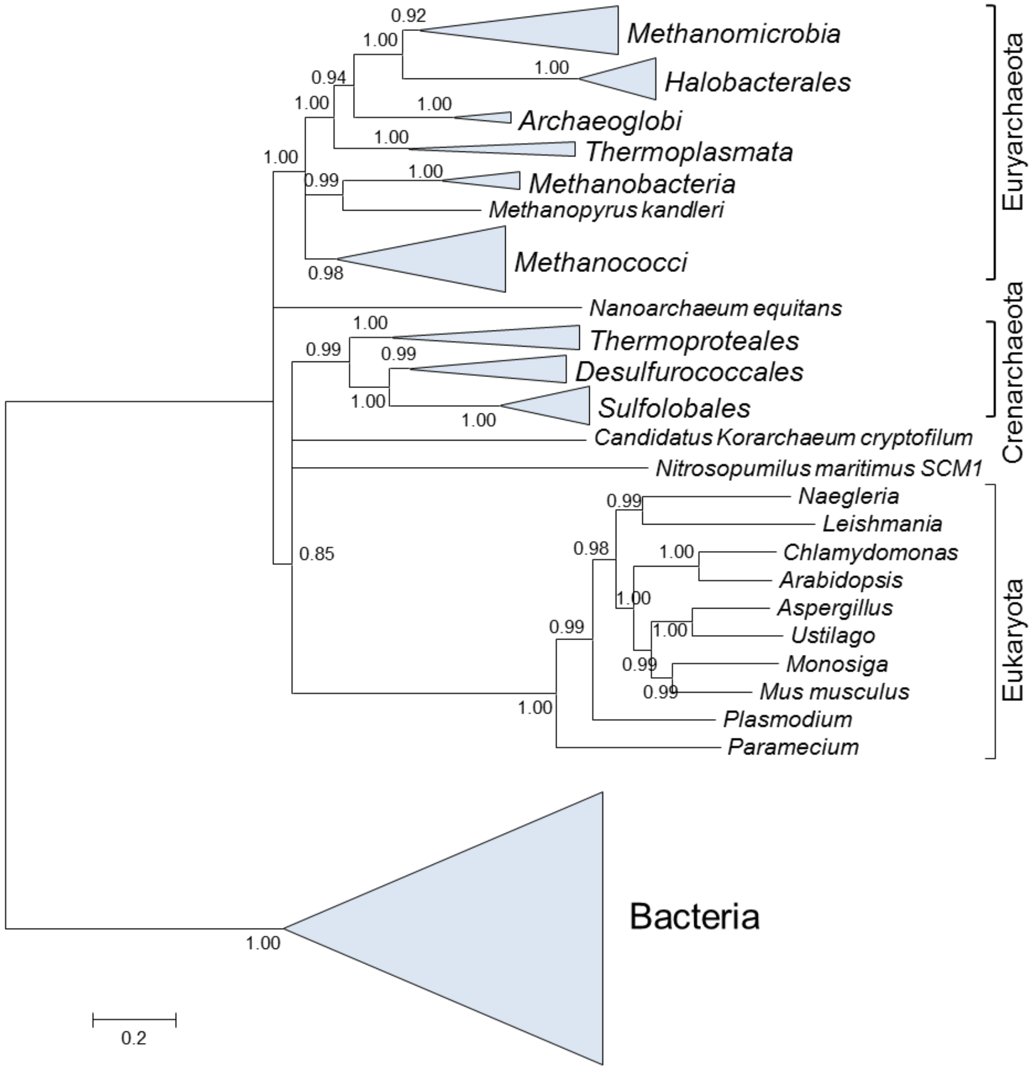

.

(((((Deinococcus:0.28502,Thermus:0.22549)1.000:0.29232,(Rubrobacter:0.41590,(Eggerthella:0.35316,(Acidimicrobium:0.37170,Frankia:0.30766)1.000:0.10034)0.958:0.05082)0.949:0.05684)0.932:0.03571,((Gloeobacter:0.25110,((Prochlorococcus:0.15372,Synechococcus:0.09196)1.000:0.21652,(Acaryochloris:0.16118,(Nostoc:0.14097,Trichodesmium:0.16784)1.000:0.04621)0.999:0.04435)1.000:0.10589)1.000:0.28404,(Dehalococcoides:0.51383,((Herpetosiphon:0.25155,Roseiflexus:0.19623)1.000:0.12586,(Thermobaculum:0.26511,(Sphaerobacter:0.14845,Thermomicrobium:0.20015)1.000:0.13144)0.972:0.04920)0.964:0.05061)1.000:0.07716)0.850:0.03507)0.552:0.02774,Aminobacterium:0.44735)0.545:0.02608,((Sebaldella:0.46992,((Staphylococcus:0.19128,(Lactobacillus:0.23004,Streptococcus:0.18456)1.000:0.07678)1.000:0.08467,(Onion:0.46557,(Mesoplasma:0.37098,(Mycoplasma:0.48367,Ureaplasma:0.52759)0.999:0.10749)1.000:0.10517)1.000:0.06770)1.000:0.09231)0.959:0.05556,((Halothermothrix:0.25106,Natranaerobius:0.27659)0.999:0.06657,(Heliobacterium:0.25843,Thermoanaerobacter:0.22685)0.877:0.04242)1.000:

0.04171)1.000:0.05087,(((Deferribacter:0.33856,Thermodesulfovibrio:0.36432)0.999:0.06497,((
Aquifex:0.29336,Persephonella:0.24277)1.000:0.20659,(Dictyoglomus:0.35448,(Coprothermoba
cter:0.42947,Fervidobacterium:0.36400)0.746:0.06560)0.837:0.05392)1.000:0.05152)0.876:0.03
966,(((((Neisseria:0.19858,(Nitrosomonas:0.29905,((Sideroxydans:0.17546,(Thiobacillus:0.1477
7,Methylovorus:0.15331)0.279:0.02629)0.314:0.01991,(Dechloromonas:0.15010,Bordetella:0.21
255)0.916:0.04016)0.942:0.03276)0.999:0.05073)1.000:0.11280,(((Francisella:0.32958,(Azotob
acter:0.19849,(Shewanella:0.12406,(Aeromonas:0.09341,(Actinobacillus:0.09903,Escherichia:0.
06342)1.000:0.05935)0.880:0.03122)1.000:0.10738)0.999:0.05071)0.992:0.02984,(Nitrosococcu
s:0.24599,Coxiella:0.32867)0.911:0.05115)0.579:0.02211,(Xanthomonas:0.29408,Dichelobacter
:0.32655)0.992:0.05371)1.000:0.04912)1.000:0.20071,(Magnetococcus:0.30527,(Rhodospirillu
m:0.21991,(Erythrobacter:0.28370,(Caulobacter:0.24272,Xanthobacter:0.19387)0.996:0.05540)1
.000:0.06210)1.000:0.17518)1.000:0.07722)1.000:0.09062,(Desulfomicrobium:0.45136,(Borreli
a:0.58285,Nautilia:0.50869)1.000:0.09579)0.346:0.03439)0.999:0.04739,((Acidobacterium:0.26
284,Solibacter:0.26036)1.000:0.27718,((((Chlorobium:0.12083,Prosthecochloris:0.11440)1.000:
0.30372,(Cytophaga:0.26793,(Bacteroides:0.25776,Blattabacterium:0.53447)0.992:0.07399)1.00
0:0.20639)1.000:0.11659,(Fibrobacter:0.53938,Gemmatimonas:0.44557)0.757:0.06543)1.000:0.
07246,(Elusimicrobium:0.62506,((Pirellula:0.62723,(((((((18976372:0.01949,(14518450:0.0128
9,14589963:0.02105)0.997:0.01399)1.000:0.04646,(242397997:0.11436,(212223144:0.02967,(2
40102057:0.02802,57639935:0.02878)1.000:0.01982)1.000:0.04732)0.998:0.03034)1.000:0.248
96,((296108688:0.08872,(261402131:0.04314,(256809973:0.01931,(15668172:0.00771,289191
496:0.00904)0.901:0.00683)0.391:0.01003)1.000:0.03278)1.000:0.10122,(150400439:0.12669,(
297618528:0.10674,(150398760:0.06125,(45357563:0.01299,(134045046:0.01009,(150401930:
0.00561,159904396:0.00699)1.000:0.00940)0.213:0.00638)1.000:0.03729)1.000:0.05265)1.000:
0.07566)1.000:0.14750)1.000:0.19264)0.981:0.04251,(20093440:0.33088,((15678031:0.02053,3
04313778:0.01273)1.000:0.09097,(84488831:0.21756,(148642060:0.11665,288559258:0.10374)
1.000:0.08806)1.000:0.06149)1.000:0.23624)0.994:0.05670)0.355:0.03244,(((288930407:0.1025
2,(11497621:0.14631,284161128:0.10899)0.816:0.03906)1.000:0.24041,(((124484829:0.28976,(
126177952:0.17881,(88601322:0.22542,(154149549:0.16635,219850687:0.15935)0.992:0.0484
7)0.579:0.03710)0.684:0.05002)1.000:0.22212,((147918682:0.11900,282162670:0.13939)1.000:
0.23051,(116753325:0.33263,((73667559:0.04584,(20088899:0.02098,21226102:0.02632)0.992
:0.01919)1.000:0.15002,(298673978:0.21289,(294494690:0.16379,91772082:0.13493)0.998:0.0
4350)0.995:0.05552)1.000:0.14510)0.906:0.04355)0.980:0.04344)0.920:0.04141,((15789340:0.0
0014,169235049:0.00014)1.000:0.14172,((222478439:0.12547,(110666976:0.13157,292654178:
0.08990)1.000:0.03844)1.000:0.05062,((76800655:0.12733,(257051090:0.11519,(257386085:0.
07667,55376942:0.08181)0.999:0.03855)1.000:0.04028)0.999:0.03132,(300709370:0.13514,(28
4163295:0.03838,289579696:0.04807)1.000:0.09771)0.715:0.02427)0.855:0.02384)0.283:0.027
54)1.000:0.42232)1.000:0.11677)0.946:0.04749,(289595678:0.23846,(48477072:0.23769,(1354
0831:0.08485,16081186:0.08188)1.000:0.18132)1.000:0.29574)1.000:0.18145)1.000:0.06713)1.
000:0.06847,38349555:0.72672)0.000:0.00742,((161527512:0.81509,(170289627:0.62703,(((14
6302785:0.20701,((15896971:0.02144,(229583573:0.00071,(((229577818:0.00023,229580722:0
.00164)0.880:0.00047,(284996407:0.00024,227829020:0.00071)0.885:0.00047)0.995:0.00211,(
227826411:0.00070,238618479:0.00014)0.000:0.00016)0.894:0.00070)1.000:0.02599)1.000:0.1
4426,(24473558:0.10913,70605853:0.14975)1.000:0.07099)0.093:0.03563)1.000:0.26540,(((126
464913:0.02102,297526028:0.02368)1.000:0.13736,(218883314:0.14438,296241748:0.13702)1.
000:0.10407)1.000:0.14539,((118430835:0.22071,302347802:0.32764)1.000:0.12897,(1240269
06:0.21576,156936795:0.31138)0.604:0.04149)0.933:0.04174)0.999:0.04969)1.000:0.09469,(11

9718918:0.37095,(159040592:0.32485,(126458628:0.05979,((145590267:0.07035,18311643:0.04283)1.000:0.02107,(119871520:0.06619,171184485:0.06290)0.983:0.01948)1.000:0.02579)1.000:0.23306)1.000:0.19034)1.000:0.10179)0.996:0.06341)0.159:0.03876)0.000:0.03409,(Paramecium:0.39081,(((Naegleria:0.35383,Leishmania:0.41110)0.998:0.06307,((Chlamydomonas:0.18449,Arabidopsis:0.17578)1.000:0.15365,((Aspergillus:0.18554,Ustilago:0.21802)1.000:0.09641,(Monosiga:0.25432,Mus:0.18931)0.996:0.05109)0.998:0.04065)1.000:0.04258)0.985:0.05400,Plasmodium:0.29166)0.998:0.08734)1.000:0.63067)0.852:0.04459)1.000:1.30757)0.000:0.06825,(Parachlamydia:0.57249,(Methylacidiphilum:0.45702,(Akkermansia:0.38662,Opitutus:0.48556)0.277:0.06249)1.000:0.12592)1.000:0.06371)0.908:0.03671)0.378:0.02545)0.885:0.03449)0.260:0.02547)1.000:0.03722)0.968:0.03092);

FastTree and RAxML phylogeny reconstruction of a sample of Bacteria, Archaea and Eukaryotes:

We selected 70 representative species of bacteria and 20 representative species of archaea by taking the mutually most distant branches from the corresponding full-set trees [6]. In the alignment of the 32 universal r-proteins from these 90 prokaryotic species and from 10 representative eukaryotic species sites with over 50% of gaps were removed. 100 bootstrap samples were taken from the filtered alignment and for each FastTree [1] (WAG evolutionary model, gamma-distributed site rates) and RAxML [3] (LG evolutionary model, gamma-distributed site rates) were used to reconstruct a phylogenetic tree. An extended majority rule consensus tree was reconstructed using the RAxML program for each set of trees.

The results (shown below) suggest a somewhat ambiguous support for inclusion of the Eukaryotes into the TACK superfilum of archaea (82% for the RAxML reconstruction, 54% for the FastTree reconstruction).

FastTree phylogeny reconstruction of a sample of Bacteria, Archaea and Eukaryotes:

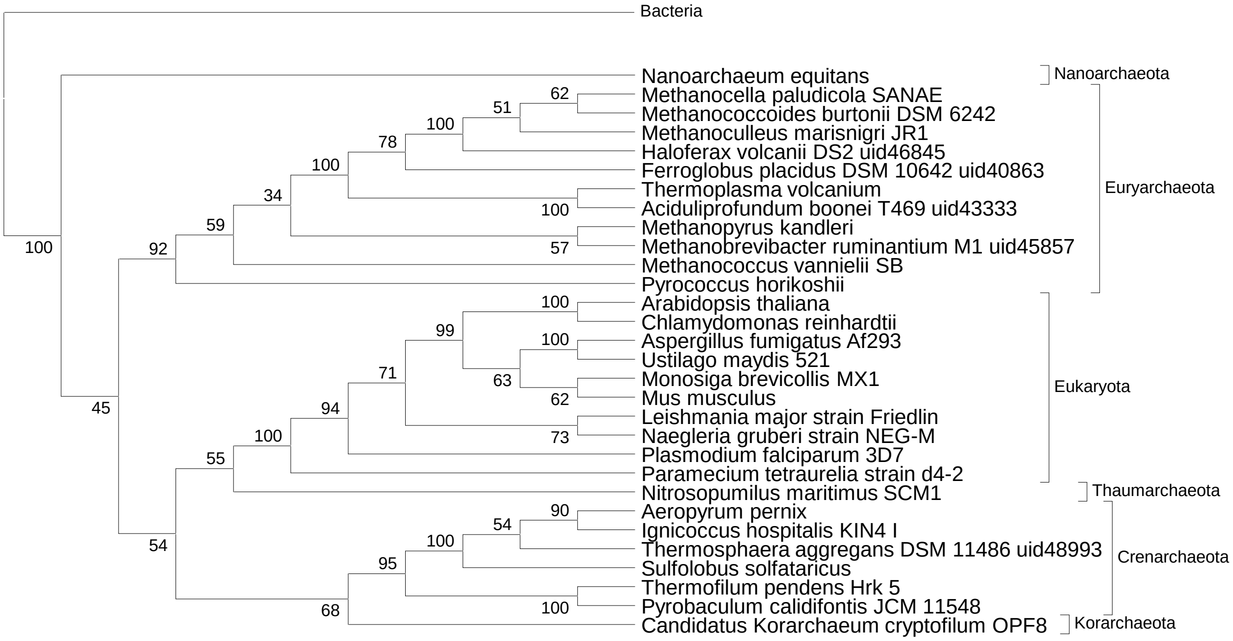

((((((((((((((Kocuria_rhizophila_DC2201,Corynebacterium_efficiens_YS-314)100,Acidimicrobium_ferrooxidans_DSM_10331)100,Cryptobacterium_curtum_DSM_15641)89,(Rubrobacter_xylanophilus_DSM_9941,Conexibacter_woesei_DSM_14684_uid43467)100)98,((Nostoc_punctiforme_PCC_73102,Prochlorococcus_marinus_MED4)100,Deinococcus_radiodurans)74)58,(((Thermobaculum_terrenum_ATCC_BAA_798,Thermomicrobium_roseum_DSM_5159)97,Chloroflexus_aggregans_DSM_9485)94,Dehalococcoides_ethenogenes_195)100)33,(((Dictyoglomus_turgidum_DSM_6724,Coprothermobacter_proteolyticus_DSM_5265)54,Kosmotoga_olearia_TBF_19_5_1)74,Aminobacterium_colombiense_DSM_12261_uid47083)49)15,(((((((Mycoplasma_genitalium,Mycoplasma_hyopneumoniae_232)99,Mycoplasma_mycoides)100,Candidatus_Phytoplasma_mali)100,(Lactobacillus_helveticus_DPC_4571,Bacillus_cereus_ATCC14579)84)76,Anaerococcus_prevotii_DSM_20548)38,(Anaerocellum_thermophilum_DSM_6725,(Clostridiales_genomosp__BVAB3_UPII9_5_uid46219,Clostridium_botulinum_A)66)53)36,((((Desulfotomaculum_reducens_MI-1,Desulfitobacterium_hafniense_Y51)97,Syntrophomonas_wolfei_Goettingen)94,(Acidaminococcus_fermentans_DSM_20731_uid43471,Symbiobacterium_thermophilum_IAM14863)58)62,(Halothermothrix_orenii_H_168,Natranaerobius_thermophilus_JW_NM_WN_LF)100)75)68)23,(Streptobacillus_moniliformis_DSM_12112,Hydrogenobaculum_Y04AAS1)22)26,(uncultured_Termite_group_1_bacterium_phylotype_Rs_D17,Elusimicrobium_minutum_Pei191)100)15,((Denitrovibrio_acetiphilus_DSM_12809_uid46657,(((Lawsonia_intracellularis_PHE_MN1-00,(Desulfotalea_psychrophila_LSv54,Desulfobacterium_autotrophicum_HRM2)92)65,(Syntrophus_aciditrophicus_SB,Geobacter_lovleyi_SZ)66)91,(Bdellovibrio_bacteriovorus,(Sorangium_cellulosum__So_ce_56,Myxococcus_xanthus_DK_1622)100)99)78)15,(Solibacter_usitatus_Ellin6076,Thermodesulfovibrio_yellowstonii_DSM_11347)31)29)18,(Campylobacter_curvus_525_92,((Methylobacterium_extorquens_AM1,(Neorickettsia_risticii_Illinois,Orientia_tsutsugamushi_Boryong)100)100,(Nitrosomonas_eutropha_C71,(Xylella_fastidiosa,Candidatus_Blochmannia_floridanus)60)100)100)47)18,((Fibrobacter_succinogenes_S85,Gemmatimonas_aurantiaca_T_27)

69,((Salinibacter_ruber_uid47323,(Bacteroides_thetaiotaomicron_VPI-5482,Blattabacterium__Blattella_germanica__Bge)100)99,Chlorobium_chlorochromatii_CaD3)100)85)24,(Leptospira_biflexa_serovar_Patoc__Patoc_1__Paris,(Borrelia_recurrentis_A1,Treponema_pallidum_SS14)100)100)34,(((Opitutus_terrae_PB90_1,Akkermansia_muciniphila_ATCC_BAA_835)53,Methylacidiphilum_infernorum_V4)100,(Pirellula_sp,Chlamydia_trachomatis_B_Jali20_OT)56)67,(((((((((Methanocella_paludicola_SANAE,Methanococcoides_burtonii_DSM_6242)62,Methanoculleus_marisnigri_JR1)51,Haloferax_volcanii_DS2_uid46845)100,Ferroglobus_placidus_DSM_10642_uid40863)78,(Thermoplasma_volcanium,Aciduliprofundum_boonei_T469_uid43333)100)100,(Methanopyrus_kandleri,Methanobrevibacter_ruminantium_M1_uid45857)57)34,Methanococcus_vannielii_SB)59,Pyrococcus_horikoshii)92,(((((((Arabidopsis_thaliana,Chlamydomonas_reinhardtii)100,((Aspergillus_fumigatus_Af293,Ustilago_maydis_521)100,(Monosiga_brevicollis_MX1,Mus_musculus)62)63)99,(Leishmania_major_strain_Friedlin,Naegleria_gruberi_strain_NEG-M)73)71,Plasmodium_falciparum_3D7)94,Paramecium_tetraurelia_strain_d4-2)100,Nitrosopumilus_maritimus_SCM1)55,(((((Aeropyrum_pernix,Ignicoccus_hospitalis_KIN4_I)90,Thermosphaera_aggregans_DSM_11486_uid48993)54,Sulfolobus_solfataricus)100,(Thermofilum_pendens_Hrk_5,Pyrobaculum_calidifontis_JCM_11548)100)95,Candidatus_Korarchaeum_cryptofilum_OPF8)68)54)45,Nanoarchaeum_equitans)100);

RAxML phylogeny reconstruction of a sample of Bacteria, Archaea and Eukaryotes:

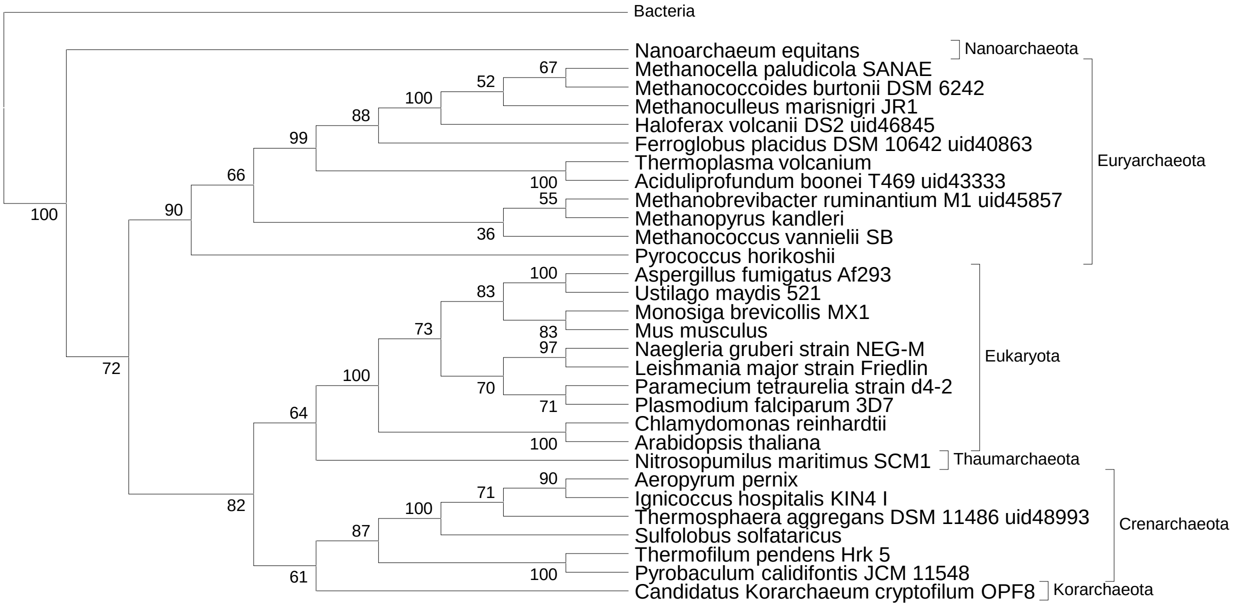

(((((((((((((((Orientia_tsutsugamushi_Boryong,Neorickettsia_risticii_Illinois)100,Methylobacterium_extorquens_AM1)100,((Candidatus_Blochmannia_floridanus,Xylella_fastidiosa)66,Nitrosomonas_eutropha_C71)100)100,(Denitrovibrio_acetiphilus_DSM_12809_uid46657,Campylobacter_curvus_525_92)55)45,(((((Desulfobacterium_autotrophicum_HRM2,Desulfotalea_psychrophila_LSv54)86,Lawsonia_intracellularis_PHE_MN1-00)62,Syntrophus_aciditrophicus_SB)45,Geobacter_lovleyi_SZ)87,(Bdellovibrio_bacteriovorus,(Sorangium_cellulosum__So_ce_56,Myxococcus_xanthus_DK_1622)100)99)78)41,(Solibacter_usitatus_Ellin6076,Thermodesulfovibrio_yellowstonii_DSM_11347)53)42,(((((Opitutus_terrae_PB90_1,Akkermansia_muciniphila_ATCC_BAA_835)42,Methylacidiphilum_infernorum_V4)100,(Pirellula_sp,Chlamydia_trachomatis_B_Jali20_OT)73)93,(Leptospira_biflexa_serovar_Patoc__Patoc_1__Paris,(Borrelia_recurrentis_A1,Treponema_pallidum_SS14)100)100)39,((((Blattabacterium__Blattella_germanica__Bge,Bacteroides_thetaiotaomicron_VPI-5482)100,Salinibacter_ruber_uid47323)98,Chlorobium_chlorochromatii_CaD3)100,(Gemmatimonas_aurantiaca_T_27,Fibrobacter_succinogenes_S85)67)98)51)24,(uncultured_Termite_group_1_bacterium_phylotype_Rs_D17,Elusimicrobium_minutum_Pei191)100)63,Streptobacillus_moniliformis_DSM_12112)20,(((((Kocuria_rhizophila_DC2201,Corynebacterium_efficiens_YS-314)100,Acidimicrobium_ferrooxidans_DSM_10331)100,Cryptobacterium_curtum_DSM_15641)90,(Rubrobacter_xylanophilus_DSM_9941,Conexibacter_woesei_DSM_14684_uid43467)100)98,((Nostoc_punctiforme_PCC_73102,Prochlorococcus_marinus_MED4)100,Deinococcus_radiodurans)74)46)18,(((((((Mycoplasma_genitalium,Mycoplasma_hyopneumoniae_232)100,Mycoplasma_mycoides)100,Candidatus_Phytoplasma_mali)100,(Lactobacillus_helveticus_DPC_4571,Bacillus_cereus_ATCC14579)63)100,Anaerococcus_prevotii_DSM_20548)84,((Clostridium_botulinum_A,Clostridiales_genomosp__BVAB3_UPII9_5_uid46219)79,Anaerocellum_thermophilum_DSM_6725)82)74,((((Desulfotomaculum_reducens_MI-1,Desulfitobacterium_hafniense_Y51)95,Syntrophomonas_wolfei_Goettingen)97,(Acidaminococcus_fermentans_DSM_20731_uid43471,Symbiobacterium_thermophilum_IAM14863)58)65,(Natranaerobius_thermophilus_JW_NM_WN_LF,Halothermothrix_orenii_H_168)99)72)100)19,

(((Thermobaculum_terrenum_ATCC_BAA_798,Thermomicrobium_roseum_DSM_5159)96,Chloroflexus_aggregans_DSM_9485)93,Dehalococcoides_ethenogenes_195)100)37,Aminobacterium_colombiense_DSM_12261_uid47083)48,(Kosmotoga_olearia_TBF_19_5_1,Dictyoglomus_turgidum_DSM_6724)63)43,Hydrogenobaculum_Y04AAS1)42,Coprothermobacter_proteolyticus_DSM_5265,((((((((Methanocella_paludicola_SANAE,Methanococcoides_burtonii_DSM_6242)67,Methanoculleus_marisnigri_JR1)52,Haloferax_volcanii_DS2_uid46845)100,Ferroglobus_placidus_DSM_10642_uid40863)88,(Thermoplasma_volcanium,Aciduliprofundum_boonei_T469_uid43333)100)99,((Methanobrevibacter_ruminantium_M1_uid45857,Methanopyrus_kandleri)55,Methanococcus_vannielii_SB)36)66,Pyrococcus_horikoshii)90,((((((Aspergillus_fumigatus_Af293,Ustilago_maydis_521)100,(Monosiga_brevicollis_MX1,Mus_musculus)83)83,((Naegleria_gruberi_strain_NEG-M,Leishmania_major_strain_Friedlin)97,(Paramecium_tetraurelia_strain_d4-2,Plasmodium_falciparum_3D7)71)70)73,(Chlamydomonas_reinhardtii,Arabidopsis_thaliana)100)100,Nitrosopumilus_maritimus_SCM1)64,(((((Aeropyrum_pernix,Ignicoccus_hospitalis_KIN4_I)90,Thermosphaera_aggregans_DSM_11486_uid48993)71,Sulfolobus_solfataricus)100,(Thermofilum_pendens_Hrk_5,Pyrobaculum_calidifontis_JCM_11548)100)87,Candidatus_Korarchaeum_cryptofilum_OPF8)61)82)72,Nanoarchaeum_equitans)100);

References.

1. Price MN, Dehal PS, Arkin AP (2010) FastTree 2--approximately maximum-likelihood trees for large alignments. PLoS One 5: e9490.
2. Darriba D, Taboada GL, Doallo R, Posada D (2011) ProtTest 3: fast selection of best-fit models of protein evolution. Bioinformatics 27: 1164-1165.
3. Stamatakis A (2006) RAxML-VI-HPC: maximum likelihood-based phylogenetic analyses with thousands of taxa and mixed models. Bioinformatics 22: 2688-2690.
4. Le SQ, Gascuel O (2008) An improved general amino acid replacement matrix. Mol Biol Evol 25: 1307-1320.
5. Matte-Tailliez O, Brochier C, Forterre P, Philippe H (2002) Archaeal phylogeny based on ribosomal proteins. Mol Biol Evol 19: 631-639.
6. Yutin N, Makarova KS, Mekhedov SL, Wolf YI, Koonin EV (2008) The deep archaeal roots of eukaryotes. Mol Biol Evol 25: 1619-1630.
7. Puigbo P, Garcia-Vallve S, McInerney JO (2007) TOPD/FMTS: a new software to compare phylogenetic trees. Bioinformatics 23: 1556-1558.
